# Exploring the determinants of AIGC usage intention based on the extended AIDUA model: a multi-group structural equation modeling analysis

**DOI:** 10.3389/fpsyg.2025.1589318

**Published:** 2025-05-21

**Authors:** Xueyan Bai, Lin Yang

**Affiliations:** ^1^School of Journalism and New Media, Xi'an Jiaotong University, Xi'an, Shaanxi, China; ^2^Research Center of New Media and Rural Revitalization, Xi'an, Shaanxi, China

**Keywords:** cognitive behavioral theory, AIDUA model, GenAI acceptance, multi-group analysis, user behavior intention

## Abstract

**Objective:**

With the rapid development and widespread adoption of generative artificial intelligence (GenAI) technologies, their unique characteristics—such as conversational capabilities, creative intelligence, and continuous evolution—have posed challenges for traditional technology acceptance models (TAMs) in adequately explaining user adoption intentions. To better understand the key factors influencing users' acceptance of GenAI, this study extends the AIDUA model by incorporating system compatibility, technology transparency, and human-computer interaction perception. These variables are introduced to systematically explore the determinants of users' intention to adopt GenAI. Furthermore, the study examines the varying mechanisms of influence across different user groups and application scenarios, providing theoretical insights and practical guidance for optimizing and promoting GenAI technologies.

**Methods:**

During the data collection phase, this study employed a survey method to measure behavioral intentions and other key variables within the proposed framework. The survey design included demographic information about the respondents as well as detailed information related to their use of GenAI. In the data processing and analysis phase, a Structural Equation Modeling (SEM) approach was utilized to systematically examine the path relationships among the variables. Additionally, to compare the differences in variable relationships across different subgroups, a multi-group structural equation modeling(MGSEM) analysis was conducted.

**Results:**

(1) Effects on Key Expectations: Social influence significantly enhances performance expectancy (β = 0.109, *p* < 0.05) but negatively impacts effort expectancy (β = −0.135, *p* < 0.01). Hedonic motivation notably mitigates effort expectancy (β = −0.460, *p* < 0.001), yet shows no significant effect on performance expectancy (β = 0.396, *p* = 0.76). The newly extended variables—technological transparency (β = 0.428, *p* < 0.001), system compatibility (β = 0.394, *p* < 0.001), and human-computer interaction perception (β = 0.326, *p* < 0.001)—demonstrate positive influences on performance expectancy while generally mitigating effort expectancy. (2) Emotional Mechanisms: Performance expectancy significantly mitigates negative emotions (β = −0.446, *p* < 0.01), while effort expectancy significantly increases negative emotions (β = 0.493, *p* < 0.001). Negative emotions exert a significant negative influence on usage intention (β = −0.256, *p* < 0.001). (3) The MGSEM analysis revealed significant heterogeneity in the extended AIDUA model paths across different user segments. Specifically, systematic variations were observed across demographic characteristics (gender, age, and educational level), occupational backgrounds, and usage patterns (task types and AI tool preferences). These findings underscore the heterogeneous nature of generative AI acceptance mechanisms across diverse user populations and usage contexts.

**Discussion:**

This study reveals several key findings within the extended AIDUA model. Our results indicate that technological transparency emerges as the strongest predictor of performance expectancy, alongside system compatibility and human-computer interaction perception, significantly enhancing users' perceived system performance. Regarding effort expectancy, hedonic motivation and technological transparency demonstrate the most prominent effects, implying that system design should emphasize user experience enjoyability and transparency. Notably, the lack of significant influence of hedonic motivation on performance expectancy, contradicting our initial hypothesis. Furthermore, the MGSEM analysis reveals significant heterogeneity in acceptance mechanisms across user groups, providing crucial implications for the differentiated design of GenAI systems tailored to diverse user needs.

## 1 Introduction

With the rapid advancement of Artificial Intelligence Generated Content (AIGC), artificial intelligence technology is profoundly transforming various facets of societal production and individual daily life (Wu et al., 2024). AIGC represents a burgeoning technology that leverages AI models to automatically generate multimodal content—including images, text, and videos—tailored to user requirements. The widespread application of this technology is not only reshaping creative production and problem-solving processes but also highlighting the extensive potential of AI technologies across a diverse range of user demographics (Setiawan et al., 2023). The launch of ChatGPT, in particular, has propelled AIGC into the limelight. Developed by OpenAI, ChatGPT is a sophisticated language generation model capable of comprehending complex human language and producing human-like conversational content. Its responses are distinguished by natural interactivity, personalization, and high accuracy. Since its release, ChatGPT has captivated societal interest and demonstrated transformative potential in numerous fields, including education, healthcare, research assistance, and content creation (Wang Y. et al., 2023).

The continuous evolution and differentiated development of AIGC tools have resulted in a diverse array of intelligent applications. ChatGPT, as an advanced natural language generation model, excels at providing engaging conversational interfaces, thereby enhancing user engagement and interactivity (Naveed et al., 2023). Conversely, DeepSeek serves as an AI platform dedicated to data analysis and reasoning, prioritizing scientific reasoning and transparent data processing to offer robust logical support and decision-making assistance (Lu et al., 2024). This emphasis on transparent data logic fosters user trust and cognitive involvement. The distinct functional goals and technical characteristics of these tools may lead to divergent pathways in user acceptance (Xu et al., 2022). However, research examining the mechanisms of user acceptance and the differentiated impacts of ChatGPT and DeepSeek remains limited, creating a theoretical gap that restricts a comprehensive understanding of how users perceive and select among various AI tools and the behavioral mechanisms underlying such choices.

Traditional technology acceptance models, such as the Technology Acceptance Model (TAM) and the Unified Theory of Acceptance and Use of Technology (UTAUT), serve as important theoretical frameworks for studying user technology adoption behavior. Proposed by Davis, TAM predicts users' behavioral intentions to adopt a technology primarily through two core variables: perceived usefulness and perceived ease of use (Ammenwerth, 2019). In contrast, UTAUT integrates multiple extended variables to enhance its generalizability, including social influence, performance expectancy, and effort expectancy, among others (Kayali and Alaaraj, 2020). These models have demonstrated high applicability in explaining user adoption behaviors for various technologies with linear functionalities, such as office software, social media tools, and mobile payment platforms (Salimon et al., 2023).

However, these traditional models exhibit several significant limitations when applied to studying artificial intelligence technologies characterized by higher complexity and dynamic features, such as AIGC tools: (1) Overlooking complex interaction experiences and anthropomorphism. TAM and UTAUT are more suited to stable technologies with straightforward functionalities, focusing on aspects like improving work efficiency or ease of use (Kim, 2014). In contrast, AIGC tools, such as ChatGPT and DeepSeek, emphasize rich human-computer interaction and anthropomorphic features as their core appeal (Chou et al., 2022). By leveraging natural language processing, these tools engage users in deep, meaningful dialogues, offering a highly interactive and anthropomorphic experience, which represents a new user experience paradigm. (2) Absence of a transparency dimension. AIGC tools often exhibit a substantial “black-box” attribute, where users are unable to clearly understand the tools' operational logic or the rationale behind their generated content (Carabantes, 2020). Transparency, as a critical dimension, plays a pivotal role in enhancing user trust and driving adoption behavior. However, neither TAM nor UTAUT incorporates transparency into their frameworks, overlooking its unique impact in the context of artificial intelligence technologies. (3) Limited focus on system compatibility. Traditional TAMs largely overlook system compatibility, despite its significance in shaping user adoption decisions. AIGC tools, characterized by diverse application scenarios, rely heavily on seamless integration across platforms, environments, and workflows, which directly influences cross-system operability and user collaboration (Liu et al., 2024). However, traditional models fail to capture the dynamic interplay between technological features and application contexts, limiting their explanatory power in the adoption of AIGC tools.

To address the aforementioned limitations and better respond to the user evaluation mechanisms for complex AI tools such as ChatGPT and DeepSeek, this study extends the AIDUA framework by innovatively introducing three key variables: system compatibility, technology transparency, and human-computer interaction perception. These variables not only enhance the theoretical multidimensional explanatory power for understanding the acceptance pathways of AIGC tools but also provide critical empirical support for optimizing the design and user experience of AI-generated technologies.

The introduction of System compatibility reflects a critical evaluation of technology acceptance theories in AIGC contexts (Ma and Huo, 2023). Drawing from the diffusion of innovations theory, system compatibility traditionally emphasizes the alignment of technology with user values. However, this approach does not fully capture the integration challenges specific to AIGC tools in complex application environments. To address this limitation, this study refines system compatibility into two dimensions: functional compatibility, which examines the alignment with workflows and user practices (Schuengel and van Heerden, 2023), and technical integration, which emphasizes interoperability and cross-platform adaptation (Vorm and Combs, 2022). These refinements offer a multidimensional framework that enhances traditional models and provides deeper insights into the adoption mechanisms of AIGC tools.

Technology transparency directly tackles the “black-box effect” inherent in AI systems, a significant challenge that undermines user trust (Harris and Blair, 2006). While existing TAMs partially address explainability, they often overlook transparency demands unique to AIGC tools. Based on research into AI trust formation (Kuhlmann et al., 2019), this study reconceptualizes transparency across two dimensions: transparency cognition, which focuses on users' understanding of the decision-making processes, and operational clarity, which highlights the predictability of system functionalities. By offering a structured framework for understanding AI transparency, this study provides critical insights into mitigating trust issues caused by system opacity.

Human-computer interaction perception bridges the gap in evaluating anthropomorphic and interactive experiences in AI systems (Wang and Qiu, 2024). Conventional models such as UTAUT fail to account for the intelligent and dynamic characteristics unique to AIGC systems. To address these shortcomings, this study refines human-computer interaction perception into two dimensions: interaction naturalness, which reflects semantic understanding and conversational fluency, and response sensitivity, which evaluates the precision and efficiency of task execution. These constructs advance the boundaries of technology acceptance research and contribute to a user-centered framework for analyzing AIGC adoption mechanisms.

In the context of AIGC, technology acceptance behavior is shaped by diverse factors, including individual characteristics (such as age and education level), usage scenarios (occupation), and task requirements (AI tool type, task type), which not only influence technology preferences and adoption willingness but also alter the strength of key factors driving acceptance pathways, thereby introducing heterogeneity in behavioral mechanisms across different user groups. To address this complexity, this study employs MGSEM to segment the sample and systematically compare the effects of key variables on adoption pathways across identified user groups. By uncovering these group-level differences, this approach provides both theoretical insights and practical guidance for designing personalized strategies to enhance the adoption of AIGC tools.

Building on these analyses, this study introduces significant advancements based on the AIDUA framework to better capture the unique adoption mechanisms of AIGC tools. The primary contributions are reflected in the following dimensions: (1) Theoretical Framework Expansion: By incorporating three critical dimensions—system compatibility, technological transparency, and human-computer interaction perception—into the AIDUA model, this study provides a refined framework to explain technology acceptance behaviors in AI-driven scenarios. These dimensions address the limitations of existing models and offer novel insights into the adoption mechanisms of AI technologies in complex application environments. (2) Differentiated Application Research: This study compares two functionally distinct AIGC tools—ChatGPT and DeepSeek—to investigate how differences in technological features shape user adoption pathways. This comparative analysis enriches the practical applications of technology acceptance frameworks within the AIGC domain and provides empirical evidence for understanding the adoption mechanisms specific to different AI tools. (3) Exploration of Individualized User Behavior: Leveraging MGSEM, this study examines the moderating effects of user characteristics—such as age, education, profession, and task type etc.—on AIGC adoption pathways. By uncovering heterogeneous mechanisms in user acceptance behavior, this research provides theoretical insights and practical strategies for personalized tool design, targeted promotion, and user experience optimization.

To achieve the research objectives, this study employed a questionnaire survey designed around core variables, targeting user groups with diverse backgrounds in age, education, and occupation to ensure representativeness. SEM was used to assess the fit of the theoretical framework and examine relationships between variables. Additionally, MGSEM explored differences in technology acceptance pathways between ChatGPT and DeepSeek, as well as group-level variations based on user characteristics.

The structure of this paper is as follows: Section 1: Introduction. This section provides a systematic overview of the research background and theoretical gap, clarifying the research questions as well as their theoretical and practical significance. Section 2: Literature Review. This section reviews existing studies from three dimensions: user adoption intentions for generative AI, TAMs, and cognitive evaluation theories, identifying theoretical gaps. Section 3: Theoretical Framework. Based on the literature review, this section constructs an integrated research model and proposes corresponding research hypotheses. Section 4: Research Design. This section details the operationalization of variables, data collection methods, and analytical strategies. Section 5: Empirical Analysis. This section presents descriptive statistics, reliability and validity tests, hypothesis testing results, and an in-depth discussion of the findings. Section 6: Research Conclusions. This section summarizes the main findings, discusses theoretical contributions and practical implications, and highlights research limitations and directions for future studies.

## 2 Literature review

### 2.1 Integration of generative AI users' acceptance willingness: a systematic literature review from the two-dimensional perspective of technical and social attributes

GenAI have developed at an unprecedented pace. Applications based on large language models (LLMs), such as ChatGPT, have significantly expanded the boundaries of human-computer interaction, driving transformative changes across various domains, including education, healthcare, and creative industries. Unlike traditional information technologies, Generative AI exhibits a high degree of autonomy, complexity, and intelligence, with technical characteristics and user interaction contexts that are notably more dynamic and flexible. These unique features have introduced unparalleled complexity to the mechanisms underlying user acceptance intentions, posing significant challenges to existing theoretical frameworks.

A systematic review of the literature reveals three key limitations in current research: First, most studies adopt a single perspective—either technological or social—to analyze user behavior, resulting in the phenomenon of “theoretical silos.” For instance, studies based on TAM primarily focus on technological functionality variables while neglecting users' socio-emotional needs. Conversely, research rooted in social cognition emphasizes interactive experiences but overlooks the foundational influence of technological usability on user decision-making (Silva, 2015). Second, there is insufficient coverage of critical variables, particularly in the emerging Generative AI context. Variables such as technology transparency, system compatibility, and human-computer interaction perception have yet to be systematically integrated into user acceptance models. Third, while traditional technology acceptance frameworks (such as TAM and UTAUT) are effective in explaining rational decision-making, they lack the necessary explanatory power to capture the complex mechanisms involving emotional, interactive, and social experiences within Generative AI environments.

To address these research gaps, this study conducts a systematic literature review, organizing existing findings along two dimensions: technological attributes and social attributes. Based on this review, an integrated analytical framework is proposed to provide a more comprehensive understanding of user acceptance mechanisms in the context of Generative AI.

In the study of user adoption intentions for GenAI, the rational perspective based on technological attributes remains one of the dominant approaches. Grounded in the TAM, the constructs of perceived usefulness and perceived ease of use are considered core factors in predicting users' acceptance behaviors (Granić and Marangunić, 2019). Research has shown that when users believe that GenAI can effectively improve productivity or learning outcomes, their adoption intentions are significantly enhanced. The UTAUT further extends these core constructs by introducing additional dimensions such as performance expectancy, effort expectancy, and facilitating conditions, all of which have been empirically verified to have strong predictive power (Legris et al., 2003).

However, the inherent complexity of GenAI presents new challenges to these traditional TAMs. Models such as the TAM and the UTAUT often fail to account for certain characteristics that distinguish GenAI from conventional technologies. For example, recent research has started to explore the impact of novel variables, including transparency and system compatibility, which are particularly relevant to the unique features of GenAI. Transparency in the context of technology refers to the degree to which information is communicated to users in an open and clear manner (Gilpin et al., 2018). This variable is particularly significant in building users' trust in the technology and in alleviating their anxieties about its use. Studies suggest that when GenAI provides users with more transparent information—such as the basis for content generation or clear explanations of algorithmic intent—adoption intentions are markedly improved (Zerilli et al., 2022). System compatibility, by contrast, emphasizes the degree to which new technologies align with users' existing technological ecosystems, workflows, and cognitive habits. The multi-scenario applications of GenAI require that it be seamlessly integrated into users' existing systems, such as office productivity tools or enterprise management platforms, to reduce usage barriers and migration costs (Bansal et al., 2019). Nonetheless, current research remains insufficient in its examination of these critical variables, particularly in the context of individual users' experiences with GenAI. This highlights the need for more in-depth studies to better understand how these emerging factors influence user adoption intentions.

The application of GenAI is influenced not only by its technological attributes but also significantly by its social attributes, which have emerged as a critical dimension in the study of user adoption intentions. Specifically, users' acceptance behaviors toward GenAI are not solely driven by rational cognition but are also closely intertwined with psychological emotions and social perceptions.

Anthropomorphism is one of the central topics in the exploration of social attributes associated with GenAI (Złotowski et al., 2015; Salles et al., 2020). Drawing from the Computers Are Social Actors theory and social response theory, it has been found that when GenAI exhibits human-like characteristics—such as emotional expression, natural tones, and responsive interactions—users perceive it as a social entity. This perception fosters emotional bonds, thereby enhancing acceptance. Research has shown that when tools such as ChatGPT incorporate warm qualities—such as humor or personalized tones—while responding to user queries, user satisfaction and trust are significantly increased (Schneider et al., 2019).

Human-Computer Interaction Perception constitutes a key social cognitive dimension shaping user adoption intentions (Diederich et al., 2022). Grounded in social presence theory and media richness theory, users' interactive experiences with GenAI directly influence their attitudes and intentions for continued use. Studies demonstrate that when users perceive the interaction to exhibit high levels of conversational coherence, context comprehension, and social reciprocity, their acceptance significantly improves. Unlike anthropomorphism, which emphasizes the human-like attributes of Artificial Intelligence, Human-Computer Interaction Perception focuses more on the quality and experience of the interaction process. It serves as a crucial bridge connecting the technological capabilities of GenAI with the psychological responses of users.

Emotional factors also play a pivotal role in the study of social attributes. Drawing from affective computing theory, users' emotional reactions during technological interactions directly regulate their adoption intentions. Positive emotions—such as satisfaction and trust—significantly enhance technology acceptance, whereas negative emotions—such as anxiety or concern about the technology—can hinder decision-making (Fernández-Batanero et al., 2021; Gelbrich and Sattler, 2014).

Additionally, Social Influence and Hedonic Motivation have garnered increasing attention for their roles in shaping user adoption intentions for GenAI (Lee et al., 2006; Inan et al., 2022). Within the frameworks of the UTAUT and the Uses and Gratifications Theory, Social Influence is recognized as a key external factor affecting users' decision-making. For example, recommendations from peers, experts, or online communities can strengthen users' positive attitudes toward GenAI (Sudirjo et al., 2023). Furthermore, GenAI can attract users by providing pleasure, enjoyment, and entertainment value. Personalized creativity and diverse expressive formats during interactions with tools like ChatGPT have been found to significantly stimulate users' intentions to adopt the technology.

Unlike existing research that adopts isolated perspectives, this study integrates both technological and social attributes, encompassing characteristics related to GenAI, and constructs a dual-faceted research framework. This study makes several key contributions to theory and methodology.

First, it develops an integrated dual-perspective framework through theoretical triangulation, unveiling the pathways through which these frameworks influence user adoption intentions. By moving beyond the limitations of a single-theory perspective, this integrated framework offers a more robust analytical tool for studying GenAI. Second, it introduces and validates critical variables—specifically, system compatibility, technological transparency, and human-computer interaction perception—and examines their varying impacts across different user groups and application scenarios. This approach enriches the contextual adaptability of acceptance theories related to GenAI. Thirdly, the theoretical contribution of this study lies in extending the boundaries of applicability for traditional TAMs to include the unique environment of GenAI, thereby addressing the limitations of traditional models in adequately explaining user acceptance in the context of GenAI.

### 2.2 The systematic evolution of the technology acceptance model: from simplistic static frameworks to complex dynamic theoretical advancements

The TAMs has undergone an evolutionary journey, moving from simplicity to complexity and from static frameworks to dynamic theoretical advancements. From the original TAM to the UTAUT, and more recently to frameworks such as the AIDUA Model, these theoretical structures have been progressively enriched and refined to better address the complexity of user behavior in new technological environments.

The TAM is a widely applied theoretical tool in the field of information behavior, designed to explore individuals' acceptance of new technologies. Grounded in the Theory of Reasoned Action, the TAM hypothesizes that two key constructs—perceived usefulness and perceived ease of use—are the primary determinants of users' intention to adopt a given technology (Bawack et al., 2023). These constructs directly influence individuals' acceptance decisions regarding new technologies and, subsequently, drive actual usage behaviors. While the original TAM is admired for its simplicity and elegance, its overly reductive assumptions have faced considerable criticism. Many scholars point out that the model overlooks the social dimensions of technology adoption and argue that relying solely on perceived usefulness and perceived ease of use is insufficient to explain the diversity of user behaviors, particularly in complex technological environments (Sok et al., 2021). This limitation becomes even more pronounced in the context of artificial intelligence devices, as these technologies often feature capabilities such as self-learning and autonomous decision-making, which add layers of complexity to user interaction. Under such circumstances, the explanatory power of the original TAM is significantly constrained.

To overcome the limitations of the TAM, the UTAUT was introduced. By retaining the core ideas of the TAM while integrating several major theoretical frameworks on technology acceptance, the UTAUT offers a more comprehensive and inclusive framework (Dwivedi et al., 2019). Among its key constructs, performance expectancy builds upon and extends the concept of perceived usefulness from the TAM, while effort expectancy originates from perceived ease of use. Additionally, the UTAUT incorporates new dimensions, such as social influence and facilitating conditions, thereby significantly broadening its explanatory scope (Choe et al., 2021). This theoretical integration enables the UTAUT to more accurately predict individuals' acceptance behaviors across diverse technological contexts.

Subsequently, the UTAUT 2 further enhanced the model's explanatory power in the domain of consumer technologies by introducing constructs such as hedonic motivation, price value, and habit (Afifa et al., 2022). These additions allowed the UTAUT 2 to place greater emphasis on consumers' intrinsic motivations, particularly the role of enjoyment and entertainment in influencing users' behavioral intentions to adopt technology. As a result, the model has been widely applied in studies on commercial and consumer-oriented technologies (Edo et al., 2023). However, despite these advancements, such models still primarily focus on traditional technology adoption contexts, limiting their applicability to explaining the adoption mechanisms of more complex technologies like artificial intelligence devices. In particular, when it comes to applications of GenAI, traditional models often fall short in capturing the impacts of key factors such as user emotions, social interactions, and the intelligent characteristics of the technology.

To address this issue, the AIDUA model represents a significant theoretical breakthrough in the field of technology acceptance research (Rudhumbu, 2022). The model's theoretical innovation lies in its departure from the static analytical frameworks of traditional TAMs. By integrating Social Response Theory and Human-Computer Interaction Theory, the AIDUA model provides a more comprehensive theoretical explanation for understanding user adoption behaviors toward artificial intelligence technologies (Schmitz et al., 2022). This model adopts a staged approach, examining user experiences across three key phases: the initial evaluation phase, the intermediate evaluation outcome phase, and the final decision-making phase. Through this framework, the model delves deeply into the process of user acceptance of GenAI technologies. Recent empirical studies have validated the predictive power of the Acceptance of AIDUA model in the context of GenAI, with particular effectiveness in explaining user adoption behaviors concerning large language model tools. These findings highlight the model's notable advantages in addressing complex adoption mechanisms unique to artificial intelligence applications.

The Acceptance of AIDUA model offers three distinct theoretical advantages: (1) Integration of AI-Relevant Variables: It enriches the traditional TAM framework by incorporating variables aligned with AI technologies, such as anthropomorphism, while retaining the traditional model's strengths. (2) Multi-Perspective Approach: The model adopts a multi-perspective framework that synthesizes both technological and social viewpoints to comprehensively explore user acceptance mechanisms. (3) The model adopts a multi-phase analytical framework, allowing for a more dynamic exploration of the mechanisms underlying the formation of user acceptance intentions. Building on these advantages, this study selects the Acceptance of AIDUA model as its theoretical foundation, further expanding its scope to achieve a more precise analysis of the user acceptance mechanisms specific to GenAI. This approach aims to offer theoretical guidance for the design and application of related products and technologies.

### 2.3 The theoretical expansion and innovation of the AIDUA model: construct integration and a multidimensional analysis framework for artificial intelligence technology acceptance

With the rapid evolution of GenAI technology, the AIDUA model has emerged as a novel tool for validating technology acceptance mechanisms, distinguished by its multidimensional explanatory capacity that integrates social, psychological, and technological factors. It has increasingly become a focal point in the field of artificial intelligence technology acceptance. This theoretical framework has been successfully extended to diverse application scenarios, including banking services, intelligent healthcare, hotel services, and financial services. In these various contexts, researchers have thoroughly examined the critical roles of existing AIDUA variables, such as social influence, hedonic motivation, and anthropomorphism, in technology acceptance. They have also conducted targeted variable expansions to more accurately analyze the technology acceptance mechanisms within each specific scenario.

In the banking services sector, scholars have expanded the explanatory boundaries of the AIDUA model by integrating technology anxiety and risk aversion, revealing that these two emotional variables significantly and negatively influence customers' willingness to accept technology (Cintamür, 2024). Moreover, in another study on sustainable banking services, an extended factor such as technology literacy was found to have a significant positive effect on customers' emotions and performance expectations (Mei et al., 2024). Research in the field of chatbots has confirmed the influence mechanisms of extended factors such as perceived novelty on technology acceptance (Ma and Huo, 2023). Further studies have demonstrated that extended cognitive factors such as personalization, competence, enthusiasm, and empathy significantly enhance consumers' willingness to accept chatbots in contactless shopping scenarios (Kim and Hur, 2024). Research in healthcare services has delved into the significance of empathy and perceived interaction quality as extended variables within the AIDUA model, highlighting their crucial facilitative roles in patients' acceptance of medical service robots (Li et al., 2024). In the hotel service sector, studies further indicate that Generation Z's willingness to accept AI devices is closely associated with the frequency of smartphone usage (Vitezić and Perić, 2021). In specific cultural contexts, such as Indian restaurant environments, extended factors such as technology familiarity have a more pronounced impact on the acceptance of service robots (Pande and Gupta, 2023). Research in educational technology has found that the characteristics of technological tasks and their alignment with task suitability are critical extended factors for students' acceptance of multimodal large language models (Al-Dokhny et al., 2024). In the realm of fintech, a study on facial recognition payment has emphasized the facilitative role of technological extension factors, such as system compatibility, on the intention to continue using such technology (Lee and Pan, 2023).

Despite the significant theoretical insights provided by the aforementioned AIDUA extension studies into the mechanisms of artificial intelligence technology acceptance, there remain critical theoretical limitations: First, existing AIDUA extension research often treats emotional responses or cognitive factors as important antecedent variables, while overlooking the fact that emotions and cognition are inherently derivative responses to artificial intelligence technology. This oversight fails to adequately illuminate the fundamental generative logic behind these emotional or cognitive variables. Second, the existing AIDUA extension studies primarily focus on model validation within specific contexts, lacking generalizability across various scenarios. Furthermore, there is insufficient elaboration on individual background characteristics, which has hindered a comprehensive understanding of the heterogeneous impacts of individual differences on the acceptance of artificial intelligence technologies.

Building upon the identified theoretical gaps, this study intends to incorporate system compatibility, technological transparency, and human-computer interaction perception into the AIDUA model as core antecedent variables. The objective is to systematically address the limitations of existing AIDUA extension research and provide a more comprehensive analytical framework for understanding the complex mechanisms of artificial intelligence technology acceptance. The specific reasons are given as follows:

Firstly, within the complex theoretical framework of technological acceptance, compared to users' cognitive and emotional factors, the fundamental attributes of technology, as an essential driving mechanism, are supported by the Evolutionary Theory of Technology Systems and Complex Adaptive Systems Theory. According to the Evolutionary Theory of Technology Systems, the inherent properties of technology not only influence user behaviors but also determine how technology continuously evolves through feedback interactions with users and their environments. The technological effectiveness and the match between technological evolution and needs constitute critical conditions for technological acceptance (Onik et al., 2017). The Complex Adaptive Systems Theory further deepens this understanding. This theory explores how the complexity and diversity of technological systems directly affect users' decision-making, adaptation strategies, and ultimately their degree of acceptance. The design, functionality, and adaptive capabilities of technology can shape users' learning approaches and feedback mechanisms during the usage process, thereby determining the extent of their acceptance and integration of new technologies. This perspective highlights the core role of technology in user adaptation and acceptance (Nan, 2011). In summary, viewing the fundamental attributes of technology as the primary driving factor and antecedent variable provides more robust theoretical support for understanding the dynamic mechanisms of technological acceptance.

Secondly, within the theoretical lineage of technological innovation, system compatibility, technological transparency, and perceived human-computer interaction constitute a multidimensional deconstruction of fundamental technological attributes. Among these, system compatibility, as a foundational attribute, provides a fundamental structural guarantee for technological acceptance. The theoretical connotation of system compatibility is rooted in the Technology-Task Matching Theory. This theory posits that within the ecosystem of technological innovation, compatibility primarily signifies the structural alignment between technological systems and specific task requirements. Such alignment transcends mere technical functional adaptation, representing a profound task-technology synergy aimed at establishing structural preconditions for technological innovation and mitigating systemic resistance to technological acceptance (Al-Rahmi et al., 2023). Technological transparency can be conceptualized as a connective attribute, with its theoretical foundation derived from Information Asymmetry Theory. Specifically, transparency plays a critical mediating role in technological systems by reducing information uncertainty and enhancing users' cognitive trust. Unlike the foundational assurance of compatibility, transparency manifests more as a connective mechanism, establishing an informational symmetry bridge between technology providers and users (Marwala and Hurwitz, 2015). Perceived human-computer interaction represents a high-order technological attribute. The human-machine interaction paradigm suggests that this attribute not only focuses on the external presentation of technological functionality but also emphasizes deep cognitive negotiation between users and technological systems. Compared to the foundational guarantee of compatibility and the connective mechanism of transparency, perceived human-computer interaction more distinctly embodies a high-order dimension of value realization, optimizing interaction pathways to enhance user experiential value (Hollender et al., 2010). In summation, these three attributes collectively constitute the fundamental properties of technology, providing a systematic theoretical perspective for understanding technological acceptance.

Furthermore, compared to the highly context-specific extended variables in existing AIDUA extension research, the three core constructs introduced in this study possess greater theoretical abstraction and generalizability. These constructs can transcend different technological ecosystems, revealing the deeper generative mechanisms of user technology acceptance behaviors, thereby providing a more macro and fundamental analytical framework for understanding the adoption of artificial intelligence technologies.

Finally, in contrast to the singular group structural equation models prevalent in existing research, this study innovatively employs a MGSEM approach to systematically examine the effects of multidimensional personal background factors—such as gender, age, education level, and occupation type—on the acceptance of artificial intelligence technologies. This methodological innovation not only addresses the theoretical limitations of existing research concerning the consideration of personal background variables but also offers a more nuanced and comprehensive analytical perspective for revealing the heterogeneity of user acceptance behaviors toward artificial intelligence technologies.

In summary, this study expands the existing AIDUA theoretical model by introducing three core constructs: system compatibility, technological transparency, and Perceived human-computer interaction. In contrast to the relatively singular and context-specific variable extensions observed in prior research, these constructs provide a more comprehensive and generalizable framework for AIDUA model expansion. This multidimensional approach systematically elucidates the complex mechanisms underlying user acceptance of artificial intelligence technologies.

### 2.4 The evolution and integration of cognitive appraisal theory: a multi-stage cognitive-affective perspective on generative AI user acceptance

The rapid rise of GenAI is reshaping the paradigms of human-computer interaction. Leveraging features such as autonomous language interaction, real-time feedback mechanisms, and adaptive learning abilities, these technologies have created interaction experiences that transcend traditional human-computer interface designs. However, to fully understand user acceptance and the intention for sustained use of GenAI, purely technology-oriented analytical frameworks are becoming inadequate. There is an urgent need to delve into the cognitive appraisal processes, emotional response mechanisms, and their respective influence pathways on user behavior and decision-making within the context of human-computer interaction. The Cognitive Appraisal Theory, as a theoretical paradigm for explaining the mechanisms of emotional formation, offers a systematic analytical framework to address these needs (Yam et al., 2021). Nevertheless, existing research based on the Cognitive Appraisal Theory faces two significant limitations. First, it struggles to effectively capture the dynamic characteristics and iterative feedback mechanisms inherent to interactions with GenAI. Second, it lacks a systematic analysis of the phased evolution of user evaluations and the interplay between cognitive and affective mechanisms that occur throughout this process. These theoretical gaps hinder a deeper understanding of the mechanisms driving user acceptance of GenAI and underscore the urgent need to construct novel evaluative frameworks tailored to these technologies.

#### 2.4.1 The theoretical origins and evolution of cognitive appraisal theory

Cognitive Appraisal Theory originated from the pioneering work of Lazarus and his colleagues in the 1960s and has since evolved from a single-dimensional appraisal model to a multidimensional appraisal framework. This theory seeks to explain how individuals evaluate environmental stimuli through subjective cognitive processes, leading to differentiated emotional responses. Unlike direct stimulus-response models, Cognitive Appraisal Theory emphasizes the mediating processes of emotional experiences, arguing that emotions are not directly triggered by external events but are instead derived from individuals' subjective evaluations of the nature and potential implications of those events.

The core appraisal structure of Cognitive Appraisal Theory comprises two interrelated yet conceptually distinct processes: (1) Primary Appraisal: This process involves an individual's evaluation of the relevance of an event to their goals (goal relevance), the congruence of the event with those goals (goal congruence), and the degree of personal involvement (ego-involvement). This stage addresses the fundamental question: “What does this event mean to me?” In the context of GenAI, primary appraisal manifests as users' evaluations of whether the capabilities of the AI are relevant to their task demands, whether they are likely to produce positive outcomes or pose potential threats, and whether they align with the users' values and sense of identity. (2) Secondary Appraisal: This process involves an individual's evaluation of their coping resources, perceived control, and expectations for future outcomes. It addresses the question: “How can I respond to this?” For users of GenAI, secondary appraisal includes assessments of their own technological competence, their ability to control AI outputs, and their expectations regarding the potential outcomes of continued interaction (Munanura et al., 2024). One of the defining characteristics of the evolution of Cognitive Appraisal Theory is its emphasis on the dynamic and context-dependent nature of the appraisal process. The theory posits that individuals' appraisal patterns adjust continuously in response to changing circumstances, interaction depth, and accumulated experiences, forming a cyclical feedback mechanism. This dynamic characteristic aligns closely with the progressive and iterative nature of interactions with GenAI, providing a robust theoretical foundation for understanding the dynamic evolution of user acceptance behaviors in this context.

#### 2.4.2 The evolution of cognitive appraisal theory in information systems research

The application of Cognitive Appraisal Theory in the field of Information Systems has undergone a notable transformation, shifting from a peripheral supplementary perspective to becoming a core analytical framework. Traditional TAMs, such as the TAM and the UTAUT, predominantly focus on cognitive factors, emphasizing rational decision-making drivers such as perceived usefulness and perceived ease of use. In contrast, the unique perspective of Cognitive Appraisal Theory, which highlights the interplay between cognition and emotion, has gradually positioned the theory as a critical complement for explaining user adoption and the sustained use of technology (Yu et al., 2023).

Compared to other cognitive-affective theories, the distinctive value of Cognitive Appraisal Theory lies in three aspects: (1) Dynamic and phased explanation of emotion formation: Unlike theories that statically address emotional outcomes, Cognitive Appraisal Theory provides a dynamic framework that captures the progression of emotions over time. (2) Emphasis on individualized subjective evaluation: The theory considers that individuals may form highly differentiated subjective evaluations of the same technological features, thereby highlighting the behavioral impact of personalized appraisals. (3) Focus on the multi-layered structure of cognitive processes: Cognitive Appraisal Theory uncovers how emotions and appraisals evolve dynamically in response to varying contexts and increased interaction depth.

While Cognitive Appraisal Theory has been widely adopted in information systems research to explain critical emotional responses such as anxiety toward technology, trust formation, and satisfaction development, its applicability to the unique domain of GenAI remains underexplored. Differing from traditional information systems, GenAI introduces an entirely novel user experience characterized by features such as real-time language interaction, autonomous learning, and creative output.

The core of this interaction experience lies in three aspects: (1) Anthropomorphic interaction design: GenAI blurs the conventional boundaries between technological tools and social partners. (2) Increased cognitive load and dynamic uncertainty: Users face higher levels of cognitive demands and unpredictability during their interactions with GenAI. (3) Deeper emotional connections: While such connections may enhance user satisfaction, they can simultaneously increase user dependency. These distinctive characteristics present significant challenges to traditional Cognitive Appraisal Theory frameworks, emphasizing the need for contextual extensions to better accommodate the complexities of GenAI user experiences.

#### 2.4.3 A multi-stage cognitive appraisal framework: an integrated model for generative AI user acceptance

**Figure 1:**
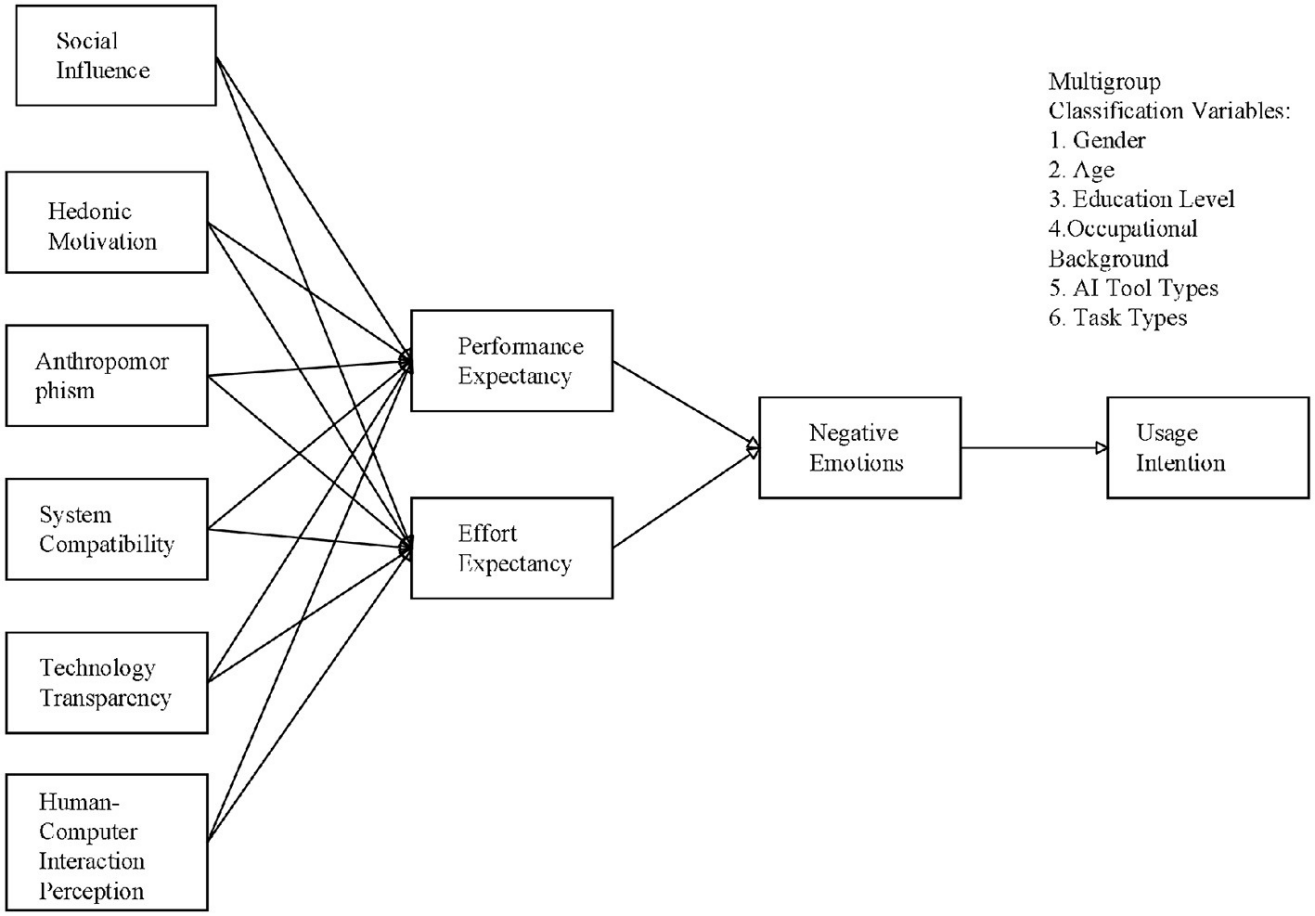
Extended AIDUA model: AIGC usage intention framework.

Building on the core concepts of Cognitive Appraisal Theory and incorporating the unique characteristics of GenAI, this study proposes a multi-stage cognitive appraisal framework that comprehensively examines the mechanisms underlying users' cognitive, emotional, and behavioral responses in GenAI use contexts (Figure 1). The framework is structured into three interrelated phases, reflecting the dynamic evolution of user experiences.

##### 2.4.3.1 Initial appraisal phase

The initial appraisal phase represents the cognitive appraisal process users undergo when first encountering GenAI. During this phase, users primarily evaluate the technology based on a rapid perception of its functional attributes and their alignment with individual needs (Wong I. K. A. et al., 2023; Milaković and Ahmad, 2023; Wang et al., 2025). Users' initial cognitive and emotional reactions are shaped by two distinct categories of factors: technological attributes and social attributes. Technological Attributes: This dimension encompasses factors such as technological transparency (the understandability of the system's functionality and underlying mechanisms) and system compatibility (the extent to which the system integrates with the user's existing technological environment and workflow). Social Attributes: This dimension includes factors such as social influence (recommendations and feedback from others), hedonic motivation (the degree of enjoyment derived from the initial interaction), human-computer interaction perception (subjective evaluations of the interaction experience), and degree of anthropomorphism (the sense of social presence and interactive affinity displayed by the system).

It is important to note that while certain appraisal factors may require some hands-on experience for accurate judgment, users often form preliminary expectations based on prior knowledge and perceptions. These early expectations serve as the foundation for a user's mental model during the initial interaction, subsequently shaping their initial appraisal outcomes and early usage intentions.

This dual-dimensional initial appraisal framework, grounded in the application of Cognitive Appraisal Theory within the GenAI context, not only highlights the theory's adaptability to this specific domain but also uncovers the complexity of early-stage cognitive formation mechanisms among users.

##### 2.4.3.2 Deep appraisal phase

The deep appraisal phase marks the point at which users engage in a more nuanced and in-depth cognitive evaluation of their interactions with GenAI (Suh, 2024; Yoon and Lee, 2021; Pei et al., 2024). From the perspective of Expectation-Confirmation Theory, the deep appraisal process is primarily driven by two core factors: performance expectations and effort expectations. Specifically, performance expectations reflect the user's evaluation of the alignment between the system's output and their intended goals, while effort expectations pertain to the user's perceived balance between operational effort and task completion efficiency.

This dynamic interplay between the dual expectations—performance and effort—directly triggers emotional responses, shaping users' experiences of cognitive load and emotional fluctuations. Throughout the deep usage phase, users compare actual system performance with their initial expectations, leading to either positive or negative emotional states. These emotional experiences, in turn, significantly influence users' long-term attitudes toward the continued use of GenAI. This framework sheds light on the cognitive-affective interaction mechanisms during deep engagement with GenAI and underscores the critical moderating role of expectations in the emotion formation process.

##### 2.4.3.3 Integrative decision phase

The integrative decision phase represents the stage where users arrive at a comprehensive evaluation of their interactions with GenAI, forming a final judgment (Chang et al., 2024). At this stage, users synthesize the cognitive assessments from the initial appraisal phase with the emotional experiences from the deep appraisal phase to develop an overarching attitude toward the use of GenAI.

When users achieve a convergence between cognition and emotion, their final usage decision is primarily driven by the cumulative impact of these prior cognitive evaluations and emotional experiences. The knowledge and emotional states accumulated through earlier phases of interaction are ultimately consolidated, translating into a user's intention to continue using GenAI.

The multi-stage cognitive appraisal framework proposed in this study makes two key contributions to the development of existing theories: (1) Addressing the limitations of traditional cognitive appraisal theory: This study constructs a multi-level model, comprising initial appraisal, deep appraisal, and integrative decision phases, by incorporating the unique characteristics of interactions with generative artificial intelligence. This effectively addresses the dimensional limitations of traditional cognitive appraisal theory in technology acceptance research. (2) Providing a Novel Theoretical Perspective for Future Research: The proposed framework offers a robust foundation for future studies to explore variations in user acceptance behaviors across different GenAI application scenarios. It also provides a basis for examining the moderating effects of individual characteristics, usage contexts, and other factors on user behavior, paving the way for further exploration of this evolving field.

## 3 Research hypotheses

### 3.1 Initial appraisal phase

In the initial appraisal phase of GenAI interactions, users' evaluations of the technology and their emotional responses are influenced by multiple factors. These factors shape users' performance expectations, effort expectations, and negative emotions through various mechanisms.

#### 3.1.1 Social influence

Both Social Cognitive Theory and the TAM emphasize that social influence is a significant external factor driving technology adoption. Social influence refers to the cognitive and behavioral tendencies individuals develop in the process of adopting technology due to the behaviors, attitudes, and normative expectations of others within their social environment (Fedorko et al., 2021; Figueroa-Armijos et al., 2023). This influence is particularly pronounced in the context of GenAI adoption, manifesting as direct social evaluation (e.g., opinions of family members, friends, or colleagues regarding the technology), indirect information dissemination (e.g., public discussions within communities or on online platforms), and norm-driven professional expectations (e.g., industry trends necessitating the use of GenAI tools). These social factors shape users' cognitive frameworks regarding GenAI, thereby directly influencing their adoption decisions (Achiriani and Hasbi, 2021; Cheng et al., 2022).

Specifically, positive social influence (e.g., high praise for GenAI within peer groups) can bolster users' confidence in the technology's functionality and value, enhancing their performance expectancy, which refers to the belief that the technology will improve task efficiency and effectiveness. On the other hand, social norms and public discourse that emphasize the complexity of the technology may increase users' perceptions of the learning burden, amplifying their effort expectancy, or the anticipated amount of effort required to use the technology effectively.

Based on the above theoretical analysis, the following hypotheses are proposed:

H1: Social influence positively affects users' performance expectancy for GenAI.

H2: Social influence negatively affects users' effort expectancy for GenAI.

#### 3.1.2 Hedonic motivation

Hedonic motivation occupies a central role in technology acceptance research, particularly in contexts where technologies exhibit a high degree of emotional interaction and entertainment attributes. Existing research has found that individuals' decisions to adopt technologies are not only driven by functional considerations but are also significantly influenced by their expectations of the pleasurable experiences the technology can provide (Siyal et al., 2021). Compared to traditional utilitarian-oriented technologies, GenAI, with its unique capacity for creative outputs, personalized interaction features, and real-time feedback mechanisms, presents tremendous potential for hedonic value, making it an ideal context for examining the influence mechanisms of hedonic motivation.

In the evaluation of GenAI applications, hedonic motivation influences users' attitude formation and behavioral intentions through two interrelated yet conceptually distinct pathways. First, when users perceive high levels of enjoyment, interactivity, or entertainment attributes in the technology, their intrinsic engagement significantly increases, thereby enhancing their confidence in and expectations of the technology's functional performance. Specifically, performance expectancy is strengthened as users perceive greater hedonic value in the technology (Mamun et al., 2024). This phenomenon aligns with the positive engagement effects highlighted in Flow Theory, which indicates that hedonic experiences can optimize users' perceptions of the practical value of the technology. Second, hedonic motivation, by increasing users' intrinsic satisfaction and emotional involvement, effectively reduces their perceived threshold for technology complexity or concerns related to cognitive load during usage. This mechanism results in a significant negative association with effort expectancy (Palos-Sanchez et al., 2024; Seng and Hee, 2021).

Based on the above theoretical analysis, this study proposes the following hypotheses:

H3: Hedonic motivation positively affects users' performance expectancy for GenAI.

H4: Hedonic motivation negatively affects users' effort expectancy for GenAI.

#### 3.1.3 Anthropomorphism

Anthropomorphism, as a core feature of technology design, plays a critical role in shaping user experiences and influencing technology acceptance. When a technological system exhibits human-like characteristics, users are more likely to perceive it as a social entity rather than a mere functional tool. In the context of GenAI applications, anthropomorphic design—by simulating human cognitive patterns and social behaviors—significantly impacts users' attitudes and emotional responses toward the technology.

Anthropomorphism influences users' acceptance of GenAI through two interrelated yet conceptually distinct pathways. First, highly anthropomorphic system designs (e.g., natural language conversations, emotionally resonant feedback, and personalized recommendations) can stimulate users' positive perceptions of the technology's intelligence. When users perceive that the technology possesses human-like capabilities, such as understanding intent, contextual reasoning, and creative thinking, they are more inclined to form optimistic expectations regarding its performance. This “anthropomorphism-trust-expectation” progression illustrates how socialized features in technology design can strengthen user confidence and significantly enhance performance expectancy (Balakrishnan et al., 2022; Polyportis and Pahos, 2025). Second, anthropomorphic features provide intuitive and natural interaction modes (e.g., contextual understanding, guided conversations, and error-tolerant mechanisms), effectively reducing the cognitive load associated with technology usage. Compared to traditional command-based or procedural interactions, anthropomorphic interactions align more closely with users' everyday social experiences, making the process of using the technology smoother and more natural. This socially oriented interaction paradigm significantly reduces users' effort expectancy, as it lowers the learning curve and simplifies the perceived complexity of using the technology (Pawlik, 2021; Yang et al., 2022). Specifically, the anthropomorphic features of GenAI—such as personalized conversational styles and intelligent feedback mechanisms—significantly enhance user engagement and trust. A high degree of anthropomorphism transforms the technology from a cold, impersonal tool into an intelligent interactive system capable of understanding, responding to, and supporting users' needs.

Based on the above analysis, this study proposes the following hypotheses:

H5: The degree of anthropomorphism positively affects users' performance expectancy for GenAI.

H6: The degree of anthropomorphism negatively affects users' effort expectancy for GenAI.

#### 3.1.4 System compatibility

According to the TAM, system compatibility is a critical determinant of users' technology adoption decisions. System compatibility refers to the degree to which a new technology aligns with users' existing values, needs, experiences, and technological environments. High compatibility reduces users' perceived risks and uncertainties, thereby facilitating the rapid diffusion of the technology. In the context of GenAI, compatibility influences user attitudes and behaviors through multiple mechanisms.

First, high compatibility enhances performance expectancy. When GenAI can seamlessly integrate with users' existing workflows—such as through open APIs for integration with office software or support for common data formats—users perceive the technology as directly improving efficiency and reducing task complexity. This “plug-and-play” characteristic minimizes cognitive load and increases users' confidence in the technology's capabilities. Moreover, alignment with users' values and preferences—such as GenAI producing outputs in styles that match user expectations—further reinforces performance expectancy (Zhang et al., 2024). Second, compatibility reduces effort expectancy. Low compatibility often results in cognitive dissonance, where the new technology conflicts with users' existing cognitive schemas, increasing learning costs and psychological stress. Conversely, high compatibility—such as when GenAI provides interfaces similar to existing tools or supports familiar interaction modalities—helps to alleviate cognitive conflicts and lower users' effort expectancy. Differences in the compatibility of various types of GenAI (e.g., text generation vs. image generation) with users' existing skills and workflows further influence effort expectancy (Shatta and Shayo, 2021).

Based on the above analysis, the following hypotheses are proposed:

H7: System compatibility positively affects users' performance expectancy for GenAI.

H8: System compatibility negatively affects users' effort expectancy for GenAI.

#### 3.1.5 Technological transparency

Technological transparency refers to the extent to which users can comprehend a system's internal mechanisms, operational principles, and decision-making processes. It is one of the critical features influencing user trust and technology adoption.

According to Technology Trust Theory, technological transparency strengthens users' trust in a technology by reducing the system's uncertainty and unpredictability, thereby shaping their attitudes and behavioral intentions. Systems with a high level of transparency allow users to clearly understand how GenAI functions and makes decisions, thereby boosting their confidence in the system's functional reliability and effectiveness, which in turn significantly enhances performance expectancy (Bodó, 2021; Wang et al., 2021). For instance, when users clearly comprehend the specific decision-making logic and data processing workflows of GenAI, they are more likely to form positive expectations regarding the system's performance. Furthermore, technological transparency can effectively reduce users' cognitive load during the process of learning and operating the system, thereby lowering their perceptions of its complexity. This cognitive simplification effect decreases the effort users perceive to be necessary for mastering the functionality of the system, thereby directly influencing their effort expectancy (Taylor et al., 2023; Durán and Jongsma, 2021). In highly transparent systems, users can more intuitively understand the system's operational principles and modes of interaction, reducing trial-and-error costs and improving overall efficiency during use. As a result, users are more likely to perceive the technology as easier to use. Conversely, systems with low transparency may lead to unclear operational logic that increases users' learning and operational efforts. Additionally, low transparency can give rise to a “black-box effect,” wherein users feel the operation of the system is opaque or unintuitive, thereby raising the perceived barriers to adoption and use.

Based on the above analysis, this study proposes the following hypotheses:

H9: Technological transparency positively affects users' performance expectancy for GenAI.

H10: Technological transparency negatively affects users' effort expectancy for GenAI.

#### 3.1.6 Perceived human-AI interaction

Perceived human-AI interaction refers to users' subjective evaluation of the quality of interaction with artificial intelligence systems. According to human-computer interaction theory, high-quality interaction strengthens users' trust in and sense of control over the system, while also increasing their engagement. This, in turn, significantly influences users' attitudes and behaviors toward the technology. In the context of GenAI, flexible and intelligent interaction designs enable users to establish an efficient and seamless communication experience with the system. Such an experience enhances users' performance expectancy (Shulner-Tal et al., 2023). For instance, when users perceive the system as having strong understanding and responsiveness, as well as the ability to flexibly manage complex tasks, they tend to develop a more favorable evaluation of the system's reliability and value. Conversely, poor interaction quality, such as sluggish system responses, inability to adapt flexibly to user needs, or low levels of intelligence in conversations, may negatively influence users' effort expectancy, which reflects their perceived complexity and cost of using the system (Chou et al., 2022). Poor interaction experiences increase the cognitive and operational effort required and may lead users to feel that the system is cumbersome or inefficient to use.

Based on this analysis, this study proposes the following hypotheses:

H11: Perceived human-AI interaction positively affects users' performance expectancy for GenAI.

H12: Perceived human-AI interaction negatively affects users' effort expectancy for GenAI.

### 3.2 Intermediate evaluation stage

#### 3.2.1 Performance expectancy

Performance expectancy refers to users' expectations regarding how technology can enhance work efficiency or task performance. In the context of technology use, users assess their emotional responses based on the extent to which a technology's performance aligns with their expectations (Zhu et al., 2024). Specifically, when the generated content meets or even exceeds user needs, this positive experience not only enhances users' trust in the AI system but also alleviates negative emotions during use. For instance, an AI-powered writing assistant that produces accurate and coherent articles can significantly reduce the user's workload while fostering positive emotional responses. However, if the system generates content that is illogical or error-prone, unmet expectations may trigger dissatisfaction, anxiety, or even lead to the abandonment of the technology.

Additionally, Cognitive Dissonance Theory also suggests that a significant discrepancy between performance expectancy and actual experiences can result in strong cognitive conflict, thereby causing emotional imbalances. When users hold high performance expectations for GenAI but find its actual performance falling short of substantially improving task outcomes, this inconsistency can magnify feelings of frustration and unease (Yin et al., 2023).

Based on this analysis, the following hypothesis is proposed:

H13: Performance expectancy negatively affects users' negative emotions.

#### 3.2.2 Effort expectation

Effort expectation refers to the perceived cognitive resources, learning costs, and operational complexity required by users to utilize a technology. It is one of the key psychological factors influencing technology adoption.

According to Cognitive Load Theory, higher effort expectation often increases users' cognitive burden, which can impact their emotional experiences (Zhang et al., 2023). Specifically, in the context of GenAI, the negative effects of effort expectation are primarily reflected in several key pathways. First, when users need to repeatedly fine-tune input commands, correct errors in AI-generated content, or understand the operational logic of the AI system, the associated learning costs and cognitive load increase significantly—leading to what is referred to as “technology fatigue” (St Omer and Chen, 2023). Second, according to the Appraisal Theory of Emotion, users' subjective assessment of the trade-off between operational cost and actual benefit during technology use directly influences their emotional responses. When users perceive the process of engaging with GenAI as overly complex, with insufficient output benefits, they are more likely to experience frustration and aversion. This evaluative process underscores the detrimental emotional effects of high effort expectation. Another critical mechanism is the trust deficit in technology. When high effort expectation leads to a reduced sense of control and comprehension regarding AI operations, users may begin to question the AI's capabilities and reliability, feeling incapable of confidently navigating the technology. This not only exacerbates technology anxiety but also triggers resistance toward the system, potentially diminishing their intention to continue using it. For instance, when users perceive that effective outcomes from GenAI heavily rely on their ability to provide highly precise and detailed input, a lack of trust in the system's responsiveness and adaptability can result in elevated psychological burdens, further destabilizing emotional equilibrium.

Based on the above analysis, the following hypothesis is proposed:

H14: Effort expectation positively affects users' negative emotions.

### 3.3 Results evaluation stage

#### 3.3.1 Negative emotions

Following the cognitive evaluations in the preceding stages, users ultimately form an overall emotional experience regarding their interaction with GenAI. According to Cognitive Appraisal Theory, emotions play a pivotal role in individual decision-making processes, particularly in the acceptance and use of new technologies (Nguyen et al., 2024). Within the context of technology use, users often rely on emotional responses as key determinants of their behavioral intentions after subjectively evaluating their interaction experiences, such as task performance or system feedback.

When users encounter unmet performance expectations, interaction barriers, or cognitive uncertainty, they may experience a range of negative emotions, including frustration, anxiety, unease, and fear. These negative emotional responses not only reduce user satisfaction but can also erode trust in the technology, undermining their willingness to adopt the system and even leading to abandonment (Peng and Hwang, 2021). In particular, Technology Threat Avoidance Theory suggests that when the perceived complexity, learning costs, or potential risks associated with a technology lead to heightened stress, users may adopt avoidance strategies to mitigate their psychological burden (Chen and Liang, 2019). This avoidance behavior directly suppresses their adoption intentions. Additionally, emotional responses play a critical mediating role by influencing users' perceptions of risk and benefit during the technology experience. Negative emotions, in particular, tend to amplify perceived risks associated with the technology while simultaneously diminishing perceived benefits, making them one of the core psychological variables shaping adoption intentions (Al-Adwan et al., 2022).

Based on the above analysis, the following hypothesis is proposed:

H15: Negative emotions negatively affect users' acceptance intention for GenAI.

## 4 Research methodology

### 4.1 Research design and questionnaire development

According to the sample size requirements for SEM, Kline, in his book Principles and Practice of Structural Equation Modeling, suggests that for complex models—such as those involving a large number of observed variables—the required sample size is typically between 10 and 20 times the number of observed variables. Following this general recommendation and considering the complexity of this study, which includes 63 observed variables to be estimated, a conservative multiplier of 15 times the number of observed variables was chosen, resulting in a minimum required sample size of 945 valid responses (Kline, 2023). To ensure data reliability and account for potential invalid responses, this study plans to collect 1,000 questionnaires, thereby further enhancing the statistical rigor and robustness of the findings.

As shown in Table 1, the questionnaire is divided into two main sections.

**Table 1:** Measurement of the structure.

**Variable name**	**Dimension**	**Item**	**Factor loading**	**Cronbach's α**	**AVE**	**CR**	**References**
Social influence	Personal influence	SI1: My friends think I should use this technology. SI2: My colleagues encourage me to use this technology. SI3: My family believes this technology is beneficial to me.	0.91, 0.83, 0.87, 0.84, 0.92, 0.86	0.83	0.76	0.93	Bagozzi et al., 2006; Liang et al., 2021
	Group influence	SI4: Information on social media motivates me to use this technology. SI5: Public opinion influences my perception of this technology. SI6: Using this technology is considered “trendy” in my social circle.					
Hedonic motivation	Pleasure	HM1: Using this technology makes me feel happy. HM2: I enjoy the process of using this technology. HM3: Using this technology is one of my ways to relax.	0.89, 0.85, 0.93, 0.82, 0.90, 0.88	0.92	0.77	0.95	Venkatesh et al., 2016; Tyrväinen et al., 2020
	Leisure	HM4: Using this technology helps me forget my worries. HM5: I use this technology as a form of entertainment. HM6: Using this technology brings me joy and pleasure.					
Anthropomorphism	Emotional interaction	AP1: I feel an emotional connection with this technology. AP2: I feel that this technology understands my emotional needs. AP3: I can establish a close relationship with this technology.	0.86, 0.81, 0.91, 0.83, 0.92, 0.85	0.87	0.76	0.94	Zhang and Rau, 2023
	Response naturalness	AP4: The responses of this technology feel as natural as those of a human. AP5: I feel the responses of this technology are warm and emotionally relatable. AP6: The feedback of this technology feels personalized.					
System compatibility	Functional compatibility	SC1: The functions of this technology are compatible with my existing devices. SC2: This technology integrates well with my current systems. SC3: Using this technology does not cause issues with my other devices.	0.90, 0.87, 0.84, 0.81, 0.93, 0.82	0.85	0.74	0.95	Al-Rahmi et al., 2021; Singh and Sinha, 2020
	Technical integration	SC4: This technology simplifies my workflow. SC5: This technology seamlessly connects with my existing devices and applications. SC6: Integrating this technology into my work/life system is effortless.					
Technology transparency	Transparency cognition	TT1: I clearly understand how this technology works. TT2: I understand how this technology processes my data. TT3: The operation of this technology is very transparent to me.	0.88, 0.80, 0.92, 0.85, 0.91, 0.83	0.86	0.75	0.94	Bai and Sarkis, 2020; Grimmelikhuijsen, 2023
	Operational clarity	TT4: I can quickly understand every function of this technology. TT5: The operation process of this technology is very clear, and I don't need to guess specific steps. TT6: I can quickly find the functions I need.					
Human-computer interaction perception	Interaction naturalness	HCI1: When using this technology, I feel its interaction style is tailored to me. HCI2: The interaction method of this technology feels smooth and intuitive. HCI3: The responses of this technology feel comfortable and meet my needs.	0.93, 0.82, 0.89, 0.86, 0.88, 0.90	0.84	0.77	0.96	Nicolescu and Tudorache, 2022; Nazar et al., 2021
	Response sensitivity	HCI4: I feel this technology responds quickly to my inputs without noticeable delay. HCI5: This technology provides timely and appropriate responses based on my specific needs. HCI6: The responses of this technology are precise and reliable.					
Performance expectancy	Efficiency improvement	PE1: Using this technology significantly improves the speed at which I complete tasks. PE2: This technology helps me save important resources (e.g., time or energy). PE3: I feel my task execution efficiency is higher with this technology.	0.87, 0.92, 0.81, 0.84, 0.85, 0.93	0.89	0.75	0.94	Vidal-Silva et al., 2024; Funmilola et al., 2019
	Task completion	PE4: This technology helps me complete tasks more easily. PE5: Using this technology allows me to successfully achieve my goals. PE6: This technology helps me achieve expected outcomes more effectively.					
Effort expectancy	Ease of use	EE1: This technology is very easy to use. EE2: I don't need much learning to use this technology. EE3: I find this technology easy to operate.	0.91, 0.88, 0.85, 0.84, 0.90, 0.86	0.88	0.76	0.95	Zhang et al., 2018; Wang et al., 2020
	Learning curve	EE4: The process of learning to use this technology is time-saving and effortless for me. EE5: This technology provides intuitive guidance to help me get started quickly. EE6: I don't feel the learning process is cumbersome or complex.					
Negative emotions	Anxiety	NE1: I feel anxious when using this technology. NE2: I feel uneasy about using this technology. NE3: I am afraid of making mistakes or being unable to use this technology correctly.	0.87, 0.92, 0.85, 0.83, 0.88, 0.91, 0.86, 0.84, 0.93	0.81	0.76	0.96	Kumar and Shah, 2021; Fan and Wang, 2022
	Frustration	NE4: I often feel frustrated when the technology fails to accurately complete my instructions. NE5: I feel depressed when dissatisfied with the overall performance of this technology. NE6: I feel helpless and frustrated when the technology does not meet my actual needs.					
	Irritation	NE7: I feel irritated when the technology fails to respond or slows down. NE8: I feel very impatient when technical failures or operational errors occur. NE9: I feel annoyed when repetitive operations are required while using the technology.					
Usage intention	Intention to continue usage	UI1: I am willing to continue using this technology. UI2: I will continue to use this technology if a new version is released. UI3: I intend to use this technology for the long term.	0.85, 0.89, 0.92, 0.90, 0.86, 0.84	0.91	0.76	0.93	Chen and Lin, 2018; Chao, 2019
	Intention to recommend	UI4: I am willing to recommend this technology to others. UI5: I will recommend this technology to my friends. UI6: I am willing to share my experience of using this technology on social media platforms.					

#### 4.1.1 Demographic variables

This section captures respondent information, including gender, age, education level, occupation type, AI tool usage experience, and the types of tasks for which AI tools are applied.

#### 4.1.2 Core construct measurement scales

This section measures key variables, including technological transparency, system compatibility, hedonic motivation, etc. All measurement items are rated on a 7-point Likert scale (1 = Strongly Disagree, 7 = Strongly Agree). Compared to a 5-point Likert scale, the 7-point scale offers higher measurement precision and greater differentiation, allowing for the capture of subtle variations in respondents' attitudes while maintaining an appropriate level of cognitive load. The 7-point scale has been widely applied and validated in psychology, behavioral sciences, and user experience research.

The measurement scales for all core variables in this study are adapted from well-established scales in classic, peer-reviewed literature (Bagozzi et al., 2006; Liang et al., 2021; Venkatesh et al., 2016; Tyrväinen et al., 2020; Zhang and Rau, 2023; Al-Rahmi et al., 2021; Singh and Sinha, 2020; Bai and Sarkis, 2020; Grimmelikhuijsen, 2023; Nicolescu and Tudorache, 2022; Nazar et al., 2021; Vidal-Silva et al., 2024; Funmilola et al., 2019; Zhang et al., 2018; Wang et al., 2020; Kumar and Shah, 2021; Fan and Wang, 2022; Chen and Lin, 2018; Chao, 2019). These scales were appropriately modified to fit the context of GenAI, detailed information is provided in Table 1.

### 4.2 Data collection and quality control

Prior to the formal survey, the research team conducted a pilot study with 30 participants using cognitive interview techniques to identify potential issues in the questionnaire design, such as ambiguous wording, logical flaws, or misunderstandings. Based on the feedback, necessary adjustments and optimizations were made to enhance the content validity and contextual suitability of the measurement instrument.

#### 4.2.1 Data collection phases

Phase One (March 2023): Participants with prior experience using ChatGPT or DeepSeek were recruited through social media platforms and university networks. This phase employed convenience sampling, a non-probability method that focuses on recruiting participants through accessible and convenient channels. To minimize selection bias, strict eligibility criteria and quality control measures were applied, ensuring that only participants meeting the research standards and having actual usage experience were included.

Phase Two (April 2023): In order to expand the coverage of the sample and enhance its representativeness, a professional survey organization was commissioned to conduct sample recruitment, employing a combination of stratified sampling and quota sampling methods. First, stratified sampling, as a probabilistic sampling method, divides the population into distinct strata based on demographic variables such as gender, age, and occupation. Quotas are then established within each stratum to ensure proportional representation of each group in the final sample. Within each quota group, random sampling is then employed to select participants. This means that individuals who meet the quota criteria are chosen randomly from all eligible candidates, ensuring fairness in participant selection.

#### 4.2.2 Inclusion criteria

To ensure the relevance and validity of the study sample, this research specifically targeted participants with experience using either ChatGPT or DeepSeek. Eligible participants were identified through two filtering questions in the questionnaire. The first question asked, “Have you ever used ChatGPT or DeepSeek?” Those who answered “No” were automatically excluded, as they did not meet the criteria for actual users. The second question required participants to describe a specific scenario in which they used these tools, allowing for further verification of their usage. Only participants providing valid responses to both questions proceeded to the survey's main sections.

#### 4.2.3 Quality control

Several quality control measures were implemented to maintain data integrity. The questionnaire included three attention-check items (e.g., “Please select ‘Strongly Disagree' for this item”) to detect inattentive responding. Responses that failed these checks were excluded. Submissions with abnormal completion times (< 1/3 or >3 × median time) and systematic response patterns (e.g., straight-lining) were also removed. Through the combination of these screening and quality control processes, participants transitioned from potential respondents to verified research participants. From the initially collected 1,000 surveys, 968 met all inclusion criteria (with a validity rate of 96.8%), providing a solid dataset for subsequent analyses.

#### 4.2.4 Common method bias

To mitigate the influence of CMB, which could arise due to the self-report nature of the questionnaire, this study implemented multiple procedural control measures during the design phase. Specifically, respondents were assured anonymity in completing the survey, items were presented in randomized order, and predictor and outcome variables were intentionally separated into different sections of the questionnaire to reduce structural biases. The questionnaire was also designed to ensure that items were simple and clear, avoiding technical jargon or double negatives to enhance response authenticity and minimize potential biases related to item wording. During the data analysis phase, Harman's single-factor test was conducted, a widely used diagnostic to assess whether CMB might be a concern. Exploratory factor analysis (EFA) revealed that the largest single factor accounted for only 26% of the total variance, significantly below the widely accepted threshold of 40%. This result confirms that no substantial common method bias was present in the data (Podsakoff et al., 2003).

Taken together, the procedural control measures adopted during the data collection phases, coupled with statistical testing during the analysis phase, ensured the reliability of the data.

### 4.3 Data analysis strategy

Data analysis was conducted using SPSS 26.0 and AMOS 24.0 software, following a two-stage approach: first, the measurement model was evaluated, and subsequently, the structural model was tested. The specific analytical steps are as follows:

First, descriptive statistical analysis was performed using SPSS to examine sample characteristics, and assess reliability through Cronbach's alpha (CA) and Composite Reliability (CR). Second, CFA was conducted using AMOS to evaluate the goodness-of-fit of the measurement model. Fit indices included χ^2^/df < 3, CFI > 0.9, TLI > 0.9, RMSEA < 0.08, SRMR < 0.08 (Marsh et al., 2004). Convergent validity was assessed through factor loadings > 0.7 and Average Variance Extracted (AVE) > 0.5 (Leguina, 2015), while discriminant validity was tested using the Fornell-Larcker criterion and the Heterotrait-Monotrait (HTMT) < 0.85 (Henseler et al., 2015). Third, SEM was employed to examine hypothesized relationships among constructs. The significance of path coefficients was assessed using the bias-corrected bootstrap method (5,000 resamples) with 95% confidence intervals. Finally, MGSEM was conducted to test potential differences in model relationships across different subgroups.

This study obtained approval from the relevant ethics committee at Xi'an Jiaotong University. All participants were informed about the research purpose and provided consent before participating in the survey. The collected data were anonymized and securely stored on an encrypted server, strictly adhering to data protection regulations.

### 4.4 Sample description

As shown in Table 2, this study collected 968 valid responses, encompassing key demographic characteristics such as gender, age, education level, occupational distribution, and AI tool usage. The age distribution revealed that the majority of respondents (38.9%) were aged 30 years or below. Overall, respondents demonstrated a high level of education, with 88.1% holding a bachelor's degree or higher. The occupational distribution highlighted that the respondents were primarily employed in knowledge-intensive industries, particularly in the information technology sector (25%) and the education sector (20%).

**Table 2:** Demographic statistics of respondents.

**Variable**	**Category**	**Number of users (N)**	**Percentage (%)**
Gender	Male	412	42.6%
	Female	556	57.4%
Age	30 years old and below	377	38.9%
	31–45 years old	320	33.1%
	46–60 years old	180	18.6%
	Above 60 years old	91	9.4%
Educational level	High school or below	116	12.0%
	Bachelor's degree	662	68.4%
	Master's degree	151	15.6%
	Doctoral degree	39	4.1%
Occupation	IT/Internet-related industries	242	25.0%
	Education/Research	194	20.0%
	Healthcare/Medical	145	15.0%
	Finance/Business Management	145	15.0%
	Manufacturing/ Engineering	126	13.0%
	Cultural media	116	12.0%
AI tool	DeepSeek	435	44.9%
	ChatGPT	533	55.1%
Task type	Creative tasks	194	20.0%
	Analytical tasks	174	18.0%
	Operational tasks	165	17.0%
	Decision-support tasks	155	16.0%
	Service/Question-answering tasks	145	15.0%
	Learning and educational tasks	135	14.0%
Total		968	100%

These characteristics align with the typical profile of current AI tool users—young, highly educated professionals predominantly working in knowledge-driven fields. Detailed demographic and occupational data are presented in Table 2.

## 5 Results

### 5.1 Measurement model evaluation

CFA was first conducted for each variable. The results showed that the unidimensional models for all variables significantly outperformed the multidimensional models and met the acceptable goodness-of-fit criteria (χ^2^/df < 3, RMSEA < 0.08, SRMR < 0.08, CFI > 0.90, TLI > 0.90). Subsequently, the evaluation of the overall model fit yielded the following results: χ^2^/df = 1.689, RMSEA = 0.037, SRMR = 0.043, CFI = 0.949, and TLI = 0.944, indicating that the model fit well (Marsh et al., 2004). Based on these findings, composite scores for each variable were calculated, and SEM approach was employed for hypothesis testing and path analysis.

As shown in Table 1, reliability analysis indicated strong internal consistency, with Cronbach's α values ranging from 0.81 to 0.92 and CR values between 0.93 and 0.96, both exceeding the recommended threshold of 0.70. In terms of convergent validity, all factor loadings were above the threshold of 0.70 (ranging from 0.80 to 0.93), and AVE ranged from 0.74 to 0.77, surpassing the standard of 0.50 (Leguina, 2015). As shown in Table 3, Discriminant validity was confirmed using both the Fornell-Larcker criterion and the Heterotrait-Monotrait ratio method. All HTMT values were below the threshold of 0.85 (Henseler et al., 2015).

**Table 3:** HTMT correlation matrix.

**Variable**	**F1**	**F2**	**F3**	**F4**	**F5**	**F6**	**F7**	**F8**	**F9**	**F10**
F1	1									
F2	0.34	1								
F3	0.47	0.38	1							
F4	0.59	0.56	0.62	1						
F5	0.63	0.51	0.48	0.67	1					
F6	0.41	0.44	0.53	0.49	0.65	1				
F7	0.72	0.75	0.68	0.74	0.77	0.71	1			
F8	0.31	0.26	0.35	0.33	0.36	0.32	0.43	1		
F9	0.23	0.29	0.37	0.28	0.46	0.21	0.27	0.25	1	
F10	0.71	0.73	0.64	0.79	0.84	0.66	0.81	0.54	0.63	1

F1–F10 represent the constructs Social Influence (F1), Hedonic Motivation (F2), Anthropomorphism (F3), System Compatibility (F4), Technology Transparency (F5), Human-Computer Interaction Perception (F6), Performance Expectancy (F7), Effort Expectancy (F8), Negative Emotions (F9), and Usage Intention (F10), with values indicating HTMT for measuring discriminant validity.

In summary, the measurement model exhibited strong reliability, validity, and fit, providing a robust foundation for subsequent structural analyses.

### 5.2 Hypothesis testing results

This study employed Structural Equation Modeling to comprehensively test the hypothesized relationships. Table 4 summarizes the results of the path analyses. Of the 15 hypotheses proposed, 14 were supported by the data, while 1 was not confirmed. The detailed analysis is as follows.

**Table 4:** Path coefficients and hypothesis testing results.

**Path**	**Path coefficient**	***p*-value**	**Result**
H1: Social Influence → Performance Expectancy	0.109	^*^	Supported
H2: Social Influence → Effort Expectancy	−0.135	^**^	Supported
H3: Hedonic Motivation → Performance Expectancy	0.396	0.760	Not supported
H4: Hedonic Motivation → Effort Expectancy	−0.460	^***^	Supported
H5: Anthropomorphism → Performance Expectancy	0.364	^***^	Supported
H6: Anthropomorphism → Effort Expectancy	−0.211	^**^	Supported
H7: System Compatibility → Performance Expectancy	0.394	^***^	Supported
H8: System Compatibility → Effort Expectancy	−0.127	^***^	Supported
H9: Technology Transparency → Performance Expectancy	0.428	^***^	Supported
H10: Technology Transparency → Effort Expectancy	−0.425	^***^	Supported
H11: Human-Computer Interaction Perception → Performance Expectancy	0.326	^***^	Supported
H12: Human-Computer Interaction Perception → Effort Expectancy	−0.225	^***^	Supported
H13: Performance Expectancy → Negative Emotions	−0.446	^**^	Supported
H14: Effort Expectancy → Negative Emotions	0.493	^**^	Supported
H15: Negative Emotions → Usage Intention	−0.256	^***^	Supported

^***^Indicates *p* < 0.001, ^**^indicates *p* < 0.01, and ^*^indicates *p* < 0.05.

#### 5.2.1 Effects of external factors on user expectations

##### 5.2.1.1 Social influence and user expectations

Social influence had a significant positive effect on performance expectancy (β = 0.109, *p* < 0.05) and a significant negative effect on effort expectancy (β = −0.135, *p* < 0.01), supporting hypotheses H1 and H2. These findings suggest that users' perception of how their social reference groups view GenAI tools not only increases their expectations of the system's performance but also alleviates concerns about usage complexity to some extent.

##### 5.2.1.2 Hedonic motivation and user expectations

The analysis indicated that hedonic motivation did not exert a statistically significant effect on performance expectancy (β = 0.396, *p* = 0.76), and thus H3 was not supported. However, hedonic motivation demonstrated a significant negative effect on effort expectancy (β = −0.460, *p* < 0.001), supporting H4. This suggests that while users' enjoyment derived from using GenAI tools might not notably enhance their expectations of the system's capabilities, it significantly reduces their perceived complexity while interacting with the tool.

##### 5.2.1.3 Anthropomorphism and user expectations

Anthropomorphic features significantly positively influenced performance expectancy (β = 0.364, *p* < 0.001) and significantly negatively influenced effort expectancy (β = −0.211, *p* < 0.01), supporting hypotheses H5 and H6. These results indicate that when GenAI tools are designed with anthropomorphic qualities, they not only enhance users' positive expectations of system performance but also reduce concerns about operational complexity.

#### 5.2.2 Effects of system characteristics on user expectations

##### 5.2.2.1 System compatibility and user expectations

System compatibility had a significant positive effect on performance expectancy (β = 0.394, *p* < 0.001) and a significant negative effect on effort expectancy (β = −0.127, *p* < 0.001), supporting hypotheses H7 and H8. These findings highlight the importance of compatibility between GenAI tools and users' existing workflows in shaping their expectations of system functionality and in alleviating operational resistance.

##### 5.2.2.2 Technical transparency and user expectations

Technical transparency exhibited a strong positive effect on performance expectancy (β = 0.428, *p* < 0.001) and a significant negative effect on effort expectancy (β = −0.425, *p* < 0.001), supporting hypotheses H9 and H10. These results underscore that when users have a higher understanding of the decision-making logic and operational transparency of GenAI tools, their confidence in system performance increases significantly while their cognitive burden during use is substantially reduced.

##### 5.2.2.3 Perceived human-computer interaction and user expectations

Perceived human-artificial intelligence interaction showed a dual influence on expectations: a significant positive effect on performance expectancy (β = 0.326, *p* < 0.001) and a significant negative effect on effort expectancy (β = −0.225, *p* < 0.001), supporting hypotheses H11 and H12. This indicates that when users perceive their interaction with GenAI as natural and smooth, it not only boosts their confidence in the system's functional performance but also reduces resistance stemming from complex interactions.

#### 5.2.3 Relationships among user expectations, emotions, and behavioral intentions

##### 5.2.3.1 User expectations and negative emotions

Results showed that performance expectancy significantly negatively affected negative emotions (β = −0.446, *p* < 0.01), while effort expectancy significantly positively influenced negative emotions (β = 0.493, *p* < 0.001), supporting hypotheses H13 and H14. These findings suggest that higher expectations of GenAI's technical performance help alleviate potential negative emotions, whereas a strong focus on operational complexity tends to amplify such negative emotions.

##### 5.2.3.2 Negative emotions and behavioral intentions

Negative emotions had a significant negative effect on behavioral intention (β = −0.256, *p* < 0.001), supporting hypothesis H15. This indicates that when users experience negative emotions (such as anxiety or unease) during their interaction with GenAI tools, their willingness to continue using the tool is significantly diminished.

#### 5.2.4 Path effect analysis

A comparison of path coefficients revealed that technical transparency (β = 0.428) emerged as the strongest positive predictor of performance expectancy. For effort expectancy, hedonic motivation (β = −0.460) and technical transparency (β = −0.425) were identified as the strongest negative predictors, effectively reducing perceived complexity. These results highlight two critical design directions for GenAI tools: enhancing technical transparency and optimizing user experience.

Overall, the majority of the hypotheses proposed in this study were supported, providing systematic evidence and empirical validation for understanding the process of user acceptance of GenAI technologies. Table 4 presents the detailed path coefficients, significance levels, and hypothesis testing results.

### 5.3 Multi-group analysis

The acceptance mechanisms of GenAI tools may exhibit significant differences across various user groups, making a single model inadequate to capture this complex heterogeneity. Particularly in the context of GenAI, which features intelligence, interactivity, and adaptability, factors such as users' technological literacy, professional background, and usage scenarios may collectively shape unique acceptance pathways. Identifying these differences is critical for both theoretical development and practical applications, thus conducting a multi-group analysis is necessary.

Before performing the multi-group analysis, this study assessed the measurement invariance of various demographic variables (such as gender, age, and educational background etc.) through configural invariance, metric invariance, and structural invariance tests to ensure the consistency and validity of the measurement tools across different groups. Specifically, As shown in Table 5, the results of the configural invariance tests indicated that the model fit indices for all groups had a CFI >0.90 and an RMSEA below 0.08, suggesting consistency in the underlying factor structure (Marsh et al., 2004). In the metric invariance tests, by constraining the factor loadings, the results showed that ΔCFI < 0.01 and ΔRMSEA < 0.015, confirming that the measurement units are equivalent across different groups and ensuring that the measurement indicators reflect the underlying constructs consistently. The structural invariance tests further verified the stability of the model across groups, with ΔCFI and ΔRMSEA also meeting strict criteria (ΔCFI < 0.01, ΔRMSEA < 0.015). This indicates that the structural parameters are largely equivalent across different groups, and the relationships among latent variables remain consistent (Cheung and Rensvold, 2002). Through these three levels of invariance testing, we robustly demonstrate the cross-group reliability of the research tools, providing rigorous statistical support for subsequent multi-group analyses.

**Table 5:** Measurement-invariance test.

**Demographic variable**	**Configural invariance (CFI, RMSEA)**	**Metric invariance (ΔCFI, ΔRMSEA)**	**Structural invariance (ΔCFI, ΔRMSEA)**
Gender	CFI = 0.923 RMSEA = 0.048	ΔCFI = 0.007 ΔRMSEA = 0.004	ΔCFI = 0.003 ΔRMSEA = 0.002
Age	CFI = 0.910, RMSEA = 0.035	ΔCFI = 0.009 ΔRMSEA = 0.005	ΔCFI = 0.004 ΔRMSEA = 0.003
Educational background	CFI = 0.930 RMSEA = 0.042	ΔCFI = 0.006 ΔRMSEA = 0.003	ΔCFI = 0.002 ΔRMSEA = 0.001
Occupational background	CFI = 0.925 RMSEA = 0.049	ΔCFI = 0.008 ΔRMSEA = 0.006	ΔCFI = 0.005 ΔRMSEA = 0.004
AI tool types	CFI = 0.920 RMSEA = 0.042	ΔCFI = 0.007 ΔRMSEA = 0.002	ΔCFI = 0.004 ΔRMSEA = 0.001
Task type	CFI = 0.915 RMSEA = 0.033	ΔCFI = 0.008 ΔRMSEA = 0.005	ΔCFI = 0.003 ΔRMSEA = 0.002

#### 5.3.1 Multi-group analysis by gender

As shown in Table 6, the results revealed significant gender differences across three critical pathways.

**Table 6:** Multi-group analysis by gender.

**Path**	**Male**	**Female**
H5: Anthropomorphism → Performance Expectancy	0.289 (^**^)	0.457 (^***^)
H13: Performance Expectancy → Negative Emotions	−0.372 (^**^)	−0.543 (^***^)
H15: Negative Emotions → Usage Intention	−0.185 (^*^)	−0.287 (^***^)

^***^Indicates *p* < 0.001, ^**^indicates *p* < 0.01, ^*^indicates *p* < 0.05.

##### 5.3.1.1 The relationship between anthropomorphism and performance expectancy (H5)

The positive association between anthropomorphism and performance expectancy was notably stronger for female users (β = 0.457, *p* < 0.001) compared to male users (β = 0.289, *p* = 0.001). This finding may be attributed to women's heightened sensitivity to social and interactive elements in technological interfaces. A possible explanation is that women tend to perceive social responsiveness as a key indicator of system capability, leading them to form stronger associations between anthropomorphic features and expected performance. In contrast, men may approach technology evaluation with a more tool-oriented mindset, focusing primarily on functional attributes rather than social characteristics.

##### 5.3.1.2 The impact of performance expectancy on negative emotions (H13)

The negative relationship between performance expectancy and negative emotions was more pronounced among women (β = −0.543, *p* < 0.001) than men (β = −0.372, *p* < 0.01). This difference could be explained by the distinct cognitive-emotional processing patterns between genders. Women might rely more heavily on their performance expectations to regulate emotional responses to technology, suggesting that their confidence in system performance plays a crucial role in reducing technology-related anxiety and other negative emotions.

##### 5.3.1.3 The effect of negative emotions on behavioral intention (H15)

The negative influence of emotional responses on behavioral intentions was stronger for female users (β = −0.287, *p* < 0.001) than male users (β = −0.185, *p* = 0.03). This pattern might be explained by gender-specific decision-making processes in technology adoption. Women potentially integrate emotional experiences more deeply into their usage decisions, while men might compartmentalize emotional responses and functional evaluations, leading to a weaker emotion-intention link in their adoption behavior.

#### 5.3.2 Multi-group analysis by age

Age, as a critical demographic variable, significantly shapes the adoption of digital technologies, particularly complex tools like GenAI. Through MGSEM. As shown in Table 7, this study identified notable age-based differences across three key pathways.

**Table 7:** Multi-group analysis by age groups.

**Path**	** ≤ 30**	**31–45**	**46–60**	**60+**
H2: Social Influence → Effort Expectancy	−0.472 (^***^)	−0.385 (^**^)	−0.294 (^*^)	−0.217 (n.s.)
H8: System Compatibility → Effort Expectancy	−0.241 (^**^)	−0.338 (^***^)	−0.412 (^***^)	−0.467 (^***^)
H12: Human-Computer Interaction Perception → Effort Expectancy	−0.183 (^*^)	−0.264 (^**^)	−0.358 (^***^)	−0.436 (^***^)

^***^Indicates *p* < 0.001, ^**^indicates *p* < 0.01, ^*^indicates *p* < 0.05, and “n.s.” indicates not significant.

##### 5.3.2.1 The effect of social influence on effort expectancy (H2)

The strength of the negative relationship between social influence and effort expectancy was highest among younger users (β = −0.472, *p* < 0.001) and weakened significantly with age, becoming non-significant for the oldest group (β = −0.217, n.s.). Contrary to assumptions that older users are more susceptible to social norms, younger users rely heavily on peer opinions and technological communities to reduce perceived difficulty, reflecting the importance of community-driven adoption mechanisms for “digital natives.” For older users, low engagement with such communities likely explains their reliance on personal judgment over external influence.

##### 5.3.2.2 The effect of system compatibility on effort expectancy (H8)

The negative relationship between system compatibility and effort expectancy became stronger with age, shifting from the younger group (β = −0.241, *p* < 0.01) to the older group (β = −0.467, *p* < 0.001). This highlights that older users rely more heavily on compatibility with their existing knowledge and habits to reduce perceived difficulty. These findings align with cognitive aging theory's “experience-dependent compensation mechanism,” which suggests older individuals adapt to new technologies by leveraging established experience templates to lower learning complexity. The results underscore the importance of system compatibility in reducing cognitive barriers for older users.

##### 5.3.2.3 The effect of perceived human-computer interaction on effort expectancy (H12)

The negative impact of human-AI interaction perception on effort expectancy intensified with age, increasing from the younger group (β = −0.183, *p* < 0.05) to the older group (β = −0.436, *p* < 0.001). Older users benefit more from high-quality interactions, such as clear responses and coherent conversations, as these features alleviate cognitive effort and uncertainty. This finding aligns with research on cognitive resource allocation, where older users, due to reduced working memory and attention, rely on immediate feedback to ease usability concerns and reduce technology-related anxiety.

These findings reveal complex generational differences in technology acceptance. Middle-aged and older users show heightened sensitivity to system compatibility and interaction quality, validating the role of cognitive aging and the “experience-dependent compensation mechanism.” In contrast, younger users emphasize social influence, illustrating the critical impact of peer evaluations and technological communities. Together, these results challenge simplistic digital divide assumptions and call for generationally adaptive GenAI designs that address varied cognitive and social needs.

#### 5.3.3 Multi-group analysis by educational background

As a key indicator of individuals' cognitive structures and critical thinking abilities, educational background can systematically influence how individuals adopt emerging technologies. To explore how variations in education levels affect the pathways of GenAI acceptance, a multi-group analysis was conducted. As shown in Table 8, the results revealed significant differences across educational levels.

**Table 8:** Multi-group analysis by education levels.

**Path**	**High school or below**	**Bachelor**	**Master**	**Doctorate**
H6: Anthropomorphism → Effort Expectancy	−0.465 (^***^)	−0.392 (^**^)	−0.274 (^*^)	n.s
H9: Technology Transparency → Performance Expectancy	n.s	0.235 (^*^)	0.384 (^***^)	0.563 (^***^)
H13: Performance Expectancy → Negative Emotions	−0.196 (^*^)	−0.287 (^**^)	−0.396 (^***^)	−0.528 (^***^)

^***^Indicates *p* < 0.001, ^**^indicates *p* < 0.01, ^*^indicates *p* < 0.05, and “n.s.” indicates not significant.

##### 5.3.3.1 The effect of anthropomorphism on effort expectancy (H6)

The negative effect of anthropomorphism on effort expectancy diminished with higher education levels. For users with high school education or below (β = −0.465, *p* < 0.001), anthropomorphism significantly reduced perceived task difficulty, while this effect became non-significant for doctoral-level users (β = −0.158, *p* > 0.05). This suggests that anthropomorphic features may be more crucial in reducing cognitive barriers for users with lower educational attainment.

##### 5.3.3.2 The effect of technical transparency on performance expectancy (H9)

The positive effect of technical transparency on performance expectancy strengthened with education levels. The relationship progressed from non-significant for high school education or below (β = 0.127, *p* > 0.05) to strongly positive for doctoral-level users (β = 0.563, *p* < 0.001). This pattern may reflect how analytical thinking skills developed through higher education enhance users' appreciation of system transparency.

##### 5.3.3.3 The effect of performance expectancy on negative emotions (H13)

The inhibitory effect of performance expectancy on negative emotions increased with education levels, from relatively weak among high school graduates (β = −0.196, *p* < 0.05) to significantly stronger among doctoral-level users (β = −0.528, *p* < 0.001). This might indicate that higher education enables users to better regulate emotions based on rational performance assessments.

These findings align with fundamental propositions of Social Cognitive Theory and Educational Cognitive Development Theory. The enhanced sensitivity to technical transparency and stronger emotional regulation among highly educated users reflects the analytical and metacognitive capabilities developed through advanced education. Meanwhile, the greater reliance on anthropomorphic features among users with lower educational attainment corresponds to Social Cognitive Theory's emphasis on how cognitive capabilities influence responses to social cues in technological interfaces.

#### 5.3.4 Multi-group analysis by occupational background

Occupational background, reflecting individuals' knowledge structure, professional needs, and technological familiarity, systematically influences the cognitive evaluation of Generative AI tools. As shown in Table 9, multi-group analysis revealed significant differences across industries in two key pathways.

**Table 9:** Multi-group analysis by industries.

**Path**	**IT and internet**	**Education and research**	**Healthcare**	**Finance and business**	**Manufacturing and engineering**	**Culture and media**
H9: Technology Transparency → Performance Expectancy	0.613 (^***^)	0.487 (^***^)	0.265 (^*^)	0.194 (^*^)	0.379 (^**^)	n.s
H11: Human-Computer Interaction Perception → Performance Expectancy	0.247 (^*^)	0.315 (^**^)	0.382 (^**^)	0.294 (^*^)	0.206 (^*^)	0.573 (^***^)

^***^Indicates *p* < 0.001, ^**^indicates *p* < 0.01, ^*^indicates *p* < 0.05, and “n.s.” indicates not significant.

##### 5.3.4.1 The effect of technical transparency on performance expectancy (H9)

Analysis revealed substantial variation across occupational groups. IT and internet professionals showed the strongest positive relationship (β = 0.613, *p* < 0.001), while cultural media practitioners demonstrated a non-significant effect (β = 0.153, *p* > >0.05). A clear pattern emerged based on technological proximity: IT and internet (β = 0.613) > education and research (β = 0.487) > manufacturing and engineering (β = 0.379) > healthcare (β = 0.265) > finance and business (β = 0.194). This pattern suggests that transparency sensitivity varies systematically with occupational characteristics. IT professionals may rely heavily on transparency for performance assessment, while cultural media practitioners potentially focus more on output quality regardless of process transparency.

##### 5.3.4.2 The effect of perceived human-computer interaction on performance expectancy (H11)

Cultural media practitioners exhibited the strongest relationship between interaction perception and performance expectancy (β = 0.573, *p* < 0.001), notably higher than manufacturing (β = 0.206, *p* < 0.05) and IT professionals (β = 0.247, *p* < 0.05). This pattern may reflect occupation-specific evaluation frameworks: cultural sectors potentially emphasize interaction quality and responsiveness, while technical sectors might prioritize functional utility. The contrast between cultural media (β = 0.573) and manufacturing (β = 0.206) suggests fundamental differences in how professions value interaction quality in technology assessment.

#### 5.3.5 Multi-group analysis by AI tool types

With the diversification of GenAI market, understanding the differences in user experience and acceptance mechanisms across various GenAI systems has become a critical research question. This study conducted a multi-group analysis focusing on users of two leading GenAI tools, DeepSeek and ChatGPT. As shown in Table 10, identified significant moderating effects of AI tool types along two core theoretical pathways.

**Table 10:** Multi-group analysis by AI tool types.

**Path**	**DeepSeek users**	**ChatGPT users**
H3: Hedonic Motivation → Performance Expectancy	0.241 (^**^)	0.389 (^***^)
H9: Technology Transparency → Performance Expectancy	0.384 (^***^)	0.235 (^*^)

^***^Indicates *p* < 0.001, ^**^indicates *p* < 0.01, ^*^indicates *p* < 0.05.

##### 5.3.5.1 Influence of hedonic motivation and technology transparency on performance expectancy (H3, H9)

In the Hedonic Motivation → Performance Expectancy pathway, the effect for ChatGPT users (β = 0.389, *p* < 0.001) is significantly stronger than for DeepSeek users (β = 0.241, *p* < 0.01). ChatGPT users tend to enhance their performance expectancy through enjoyable interactions, reflecting an “experience-enhanced expectancy pattern.” The enjoyable user experience not only provides emotional satisfaction but also strengthens their expectations of the system's functional efficiency. For DeepSeek users, the influence of hedonic motivation is comparatively weaker, with performance expectancy primarily driven by the system's functional features.

In the Technology Transparency → Performance Expectancy pathway, the effect for DeepSeek users (β = 0.384, *p* < 0.001) is significantly stronger than for ChatGPT users (β = 0.235, *p* < 0.05). DeepSeek users regard transparency as a crucial factor in evaluating system trustworthiness and functional reliability, particularly for newer platforms where information asymmetry is evident. Technology transparency reduces cognitive uncertainty and provides a reliable foundation for performance expectation. On the other hand, ChatGPT users rely more on accumulated user experiences and system performance consistency, making the impact of transparency on performance expectancy relatively weaker.

This contrast highlights the distinct functional positions of the two AI systems in users' mental models: ChatGPT leverages enjoyable experiences to elevate performance expectancy, while DeepSeek builds user confidence in system capabilities through technology transparency. These results offer insights for generative AI design—platforms should balance “enjoyability vs. transparency” based on user preferences, strengthening their unique advantages to meet diverse user needs.

#### 5.3.6 Multi-group analysis by task type

This section presents the findings of multi-group SEM, which examines how key theoretical pathways differ across various types of generative AI tasks. As shown in Table 11, the results reveal distinct variations in theoretical relationships depending on task characteristics, as outlined below.

**Table 11:** Multi-group analysis by task types.

**Path**	**Creative task**	**Analytical task**	**Operational task**	**Decision support**	**Service Q&A**	**Learning and education**
H4: Hedonic Motivation → Effort Expectancy	−0.547 (^***^)	−0.236 (^*^)	n.s	n.s	−0.493 (^***^)	−0.387 (^**^)
H10: Technology Transparency → Effort Expectancy	−0.186 (^*^)	−0.562 (^***^)	n.s	−0.594 (^***^)	n.s	n.s
H13: Performance Expectancy → Negative Emotions	−0.214 (^*^)	−0.497 (^***^)	n.s	−0.542 (^***^)	n.s	−0.185 (^*^)

^***^Indicates *p* < 0.001, ^**^indicates *p* < 0.01, ^*^indicates *p* < 0.05, and “n.s.” indicates not significant.

##### 5.3.6.1 The effect of hedonic motivation on effort expectancy (H4)

Hedonic motivation had a stronger effect on effort expectancy in creative tasks (β = −0.547, *p* < 0.001) and service-oriented Q&A tasks (β = −0.493, *p* < 0.001) compared to analytical tasks (β = −0.236, *p* < 0.05). This result indicates that enjoyable and engaging interactions are particularly effective at reducing perceived difficulty in process-oriented or exploratory tasks like creative writing and Q&A interactions. Conversely, in result-oriented tasks like analytical work, users tend to prioritize functional performance and are less influenced by hedonic factors in their perception of task effort.

##### 5.3.6.2 The effect of technology transparency on effort expectancy (H10)

The relationship between technology transparency and effort expectancy varied across task types. It was strongest in decision support tasks (β = −0.594, *p* < 0.001) and analytical tasks (β = −0.562, *p* < 0.001), indicating that increased transparency significantly reduced the perceived difficulty of tasks that require accurate, reliable, and accountable outputs. In creative tasks (β = −0.186, *p* < 0.05), however, the impact was weaker, reflecting the fact that creative outcomes tend to be more subjective and flexible, thereby diminishing the relative importance of transparency in reducing effort perception. These findings highlight that for tasks with high stakes and fixed outcome expectations, such as decision-making or data analysis, transparency plays a critical role in reducing cognitive workload and perceived effort. In contrast, in creative tasks where output criteria are less rigid, transparency has a weaker influence on perceived task difficulty.

##### 5.3.6.3 The effect of performance expectancy on negative emotions (H13)

The negative relationship between performance expectancy and negative emotions was strongest in decision support tasks (β = −0.542, *p* < 0.001) and analytical tasks (β = −0.497, *p* < 0.001), indicating that higher performance expectancy significantly reduced negative emotional responses in tasks with rigid, well-defined outcome standards. On the other hand, this relationship was weaker in creative tasks (β = −0.214, *p* < 0.05) and learning tasks (β = −0.185, *p* < 0.05), where flexible and open-ended outcomes allow users greater tolerance for unmet expectations, thereby dampening the emotional impact. These findings demonstrate that tasks with fixed evaluation criteria, such as decision-making and analytical work, are more sensitive to unmet expectations, which can amplify frustration and negative emotions. In contrast, the flexible structures of creative and educational tasks allow greater adaptability and lower emotional stakes, reducing the influence of performance expectancy on negative emotional outcomes.

## 6 Discussion

### 6.1 Theoretical validation and extension of antecedent variables in the GenAI acceptance model

This study partially validates existing theoretical frameworks for AI acceptance while significantly extending their scope and applicability (Lin et al., 2022). In alignment with predictions from the traditional UTAUT model, social influence was found to have a significant positive effect on users' performance expectancy (β = 0.109, *p* < 0.05), confirming the critical role of social embeddedness in technology acceptance processes (Du et al., 2023). However, this study highlights that system characteristic variables—such as technical transparency (β = 0.428), system compatibility (β = 0.394), and perceived human-computer Interaction (β = 0.326)—exert a significantly stronger influence on performance expectancy compared to social influence. These findings empirically challenge the traditional emphasis on social influence as a central determinant in TAMs. Particularly, the dominance of technical transparency as the strongest predictor underscores that users' expectations regarding complex AI systems heavily rely on their understanding of how these systems operate. This finding highlights the critical importance of incorporating transparency into the design of high-cognitive-complexity technologies.

An important and unexpected finding is that hedonic motivation did not have a significant effect on performance expectancy (β = 0.396, *p* = 0.76), which notably deviates from prior research (Rahmiati and Susanto, 2022). This indicates that there is a unique cognitive mechanism in the acceptance of GenAI, where users are able to clearly distinguish between the system's functional value and the pleasure derived from its use. This phenomenon is termed “function-hedonic decoupling,” which may stem from the fact that generative artificial intelligence possesses both tool-like and hedonic attributes, leading to a relative independence between functionality and enjoyment in evaluations.

The significant positive effect of anthropomorphism on performance expectancy (β = 0.364, *p* < 0.001) validates the critical role of anthropomorphism within AIDUA model, further reinforcing its position as a foundational variable in the theoretical framework of AI acceptance. The findings demonstrate how anthropomorphic design significantly enhances users' performance expectancy, offering new theoretical insights for AI acceptance research (Wang C. et al., 2023). The findings align with Social Response Theory, which suggests that individuals naturally apply social rules and expectations to their interactions with technology. In the context of GenAI systems, anthropomorphic features may serve as a “competence signal,” enabling users to form intuitive judgments about system capabilities through familiar social cognitive frameworks. This cognitive mechanism could explain how anthropomorphism enhances performance expectations while simultaneously reducing perceived operational complexity.

This study verifies a dual moderating mechanism of system characteristics on user expectations. Empirical evidence indicates that system characteristics (e.g., technical transparency, system compatibility, and perceived human-computer Interaction) exert bidirectional influences on user perceptions: significantly enhancing performance expectancy while reducing effort expectancy. This “function-effort dual moderation” phenomenon provides a more granular cognitive framework for understanding technology acceptance processes. Specifically, system characteristics not only strengthen acceptance intentions by elevating users' expectations of technological performance but also alleviate cognitive burdens during the usage process. Notably, technical transparency demonstrates the most significant bidirectional moderating effects, with remarkable influence intensities on both performance expectancy (β = 0.428) and effort expectancy (β = −0.425). This finding aligns with Cognitive Load Theory, revealing transparency's core role as a cognitive aid mechanism in complex AI systems: enhancing users' cognitive understanding of system functionality while effectively reducing perceived usage difficulty.

### 6.2 Negative emotions as a cognitive mediating mechanism between user expectations and behavioral intentions

This study validates the important role of negative emotions in the relationship between user expectations and usage intention, revealing a cognitive-affective interaction mechanism within the GenAI acceptance process (Marzouki et al., 2021). Empirical results show that performance expectancy exerts a significant negative effect on negative emotions (β = −0.446, *p* < 0.01), while effort expectancy has a significant positive predictive effect on negative emotions (β = 0.493, *p* < 0.001). Further analysis indicates that negative emotions have a significant negative influence on usage intention (β = −0.256, *p* < 0.001). This clear mediating pathway suggests that the relationship between user evaluations of the system and final behavioral decisions is not linear but rather mediated by a complex cognitive-affective interaction.

#### 6.2.1 Dual cognitive appraisal mechanism

The opposing effects of performance expectancy and effort expectancy on negative emotions highlight the existence of a dual cognitive appraisal mechanism during the technology acceptance process (Gódány et al., 2021). Users simultaneously engage in two types of evaluations: (1) a positive appraisal of the system's functional benefits (performance expectancy) and (2) a negative appraisal of usage costs or potential barriers (effort expectancy). These dual appraisals collectively influence emotional responses, illustrating users' systematic trade-offs in the context of complex technology adoption.

This mechanism aligns with cognitive appraisal theory and uncovers task-specific dimensions when applied to the technology acceptance domain (Cai and Cheng, 2024). Notably, the influence strengths of performance expectancy and effort expectancy on emotions are comparable (|β| ≈ 0.5), indicating that for high-cognitive-complexity technologies like GenAI, users weigh both the benefits and potential drawbacks of the technology to shape their emotional responses. This finding offers new cognitive insights for technology acceptance theory, emphasizing the balanced interaction between users' perceptions of gains and cognitive burdens.

#### 6.2.2 Cognitive load and the emotion-first effect

The strong positive effect of effort expectancy on negative emotions (β = 0.493) aligns closely with existing research (Gunasinghe and Nanayakkara, 2021). High-cognitive-complexity technologies such as GenAI may trigger heightened emotional stress among users due to “cognitive burden anxiety,” which arises from the black-box nature of AI systems and the uncertainty of their outputs.

Concurrent with this, the significant inhibitory effect of negative emotions on usage intention (β = −0.256) underscores the pivotal role of users' emotional experiences in shaping behavioral decisions. This “emotion-first effect” suggests that even when users harbor positive expectations for the system's functionality, negative emotional experiences during use can substantially reduce their intention to adopt the technology. This finding is consistent with the Risk-as-Feelings Theory, which posits that emotional responses can operate independently of cognitive evaluations and hold significant importance in actual decision-making processes.

#### 6.2.3 Structural shift in the evaluation framework

The study also reveals a distinctive acceptance logic for GenAI in contexts devoid of alternative human-intervention solutions. Unlike traditional AI applications, where users often compare AI systems to human services, GenAI prompts an absolute evaluation based solely on the system's capabilities. Consequently, performance expectancy and effort expectancy exhibit heightened capacities to influence emotional responses. While increases in performance expectancy significantly buffer negative emotions, heightened effort expectancy is more likely to evoke anxiety, further diminishing user adoption intentions. This paradigmatic shift in the evaluation framework underscores that, compared to hybrid human-AI systems, acceptance of standalone AI tools like GenAI relies more heavily on striking a balance between functional and emotional experiences.

By introducing negative emotions as a mediating variable, this study proposes a more systematic “Cognition-Affect-Behavior” three-stage model, addressing the long-standing oversight of emotional experience in existing technology acceptance theories. Traditional frameworks such as TAM and UTAUT emphasize a direct pathway from cognitive appraisals (e.g., performance and effort expectancies) to behavioral intentions. In contrast, this study demonstrates that emotional variables serve as a critical bridge in the transformation of user expectations into behavioral intentions.

Additionally, this study empirically validate the dual role of negative emotions in the AI acceptance process. On the one hand, negative emotions reflect users' perceived intensity of technological barriers during the experience; on the other hand, as a decision-suppressing factor, they emphasize the importance of emotional experiences in shaping technology acceptance behaviors. This mechanism extends the scope of traditional technology acceptance theories, offering a new perspective for understanding how users interact with high-cognitive-complexity technologies like GenAI.

### 6.3 Differential moderating effects of demographic characteristics on GenAI acceptance

Demographic variables exhibit systematic moderating patterns in the AI acceptance process, going beyond simple descriptive differences to uncover the deeper cognitive mechanisms and socialization processes shaping technology evaluation.

(1) The findings reveal that female users demonstrate stronger associations between anthropomorphism and performance evaluation, as well as along emotional pathways. This aligns with observations from evolutionary psychology, which suggest that women are more likely to integrate social cues with functional evaluations, whereas men tend to separate the two. This challenges the conventional practice of treating gender as a mere control variable in technology acceptance research and underscores the necessity of addressing gender-specific social and emotional needs in GenAI design.

(2) Age-related effects highlight generational differences in cognitive processing strategies. Older users exhibit a higher dependency on system compatibility and interaction quality, reflecting a cognitive resource reallocation strategy. In contrast, younger users display an unexpectedly high sensitivity to social influence, subverting traditional age-related stereotypes in technology acceptance. These findings suggest that GenAI design must move beyond the oversimplified “digital natives vs. digital immigrants” dichotomy and recognize the distinct evaluation frameworks employed by different generational groups.

(3) As a proxy for cognitive complexity, education level reveals its role in shaping multi-layered cognitive judgments of GenAI. Highly educated users show a stronger reliance on technical transparency, while less-educated users exhibit a preference for anthropomorphism. This reflects how metacognitive abilities reshape users' standards for evaluating technology. Such differentiated patterns suggest that GenAI design must strike a balance between explainability and usability, tailored to the cognitive characteristics of target user groups.

(4) Different GenAI tools significantly shape user acceptance mechanisms. DeepSeek users rely on technical transparency to build trust, reflecting a “rational compensation” mechanism, while ChatGPT users focus on system performance, aligning with “performance-oriented trust.” Furthermore, ChatGPT users show an “experience-driven” tendency through greater hedonic motivation, whereas DeepSeek users adopt a “tool-oriented” approach. These findings underscore the need for differentiated design strategies to align with user expectations and cognitive frameworks.

(5) The influence of professional background extends beyond mere skill differences, highlighting how occupational socialization cultivates specific paradigms for evaluating technology. IT professionals prioritize transparency, traditional industry workers emphasize compatibility, and creative professionals focus on interaction quality. These three distinct patterns illustrate how professional roles shape foundational frameworks for technology value judgments. This finding challenges the validity of universal design approaches and points to the need for differentiated development strategies based on occupational cognitive models.

(6) Different task types significantly influence user acceptance mechanisms in Generative AI systems. For high-stakes, outcome-critical tasks such as decision support and analytical tasks, users prioritize technology transparency to establish trust, reflecting a “risk-sensitive trust-building” mechanism. Conversely, in creative and process-oriented tasks, users focus more on interaction quality and hedonic motivation, aligning with an “engagement-driven utilization” approach. Moreover, performance expectancy plays a dominant role in closed-ended tasks, signaling a “precision-reliance” dynamic, while open-ended tasks demonstrate a greater tolerance for flexibility and an “adaptability-seeking” framework. These findings highlight the importance of tailoring system design to task-specific demands, ensuring alignment with users' cognitive priorities and task characteristics.

These differentiated moderating mechanisms not only enrich technology acceptance theories but also pose fundamental challenges for GenAI design. Future system development must move beyond function-oriented, one-size-fits-all designs and adopt precision-matching strategies based on users' cognitive characteristics. Such an approach is critical for promoting the inclusive adoption of AI technologies and ensuring equitable benefits across diverse user groups.

## 7 Research significance

### 7.1 Theoretical contributions

This study makes significant advancements in technology acceptance theory and the understanding of generative AI acceptance mechanisms, which can be summarized in three core areas.

#### 7.1.1 Extending the AIDUA model with technical characteristics

This study significantly extends the AIDUA model by introducing key variables such as technological transparency, system compatibility, and perceived human-AI interaction. While traditional AIDUA frameworks primarily emphasize the influence of social factors on AI acceptance, our findings indicate that, in complex systems such as generative AI, user acceptance is more profoundly influenced by intrinsic system characteristics rather than by external social factors. This shift from a “social reference” model to a direct evaluation of system capabilities underscores how increasing technological complexity is reshaping user evaluation frameworks.

Furthermore, this research addresses several critical theoretical gaps present in the existing AIDUA extension literature. First, we emphasize that technology should be viewed as a primary antecedent variable, proposing that emotional responses are derivative cognitive reactions arising from user interaction with the technology. This perspective clarifies the fundamental logic behind the generation of emotional variables and their intrinsic relationship with technology. Additionally, we advocate for the necessity of constructing a more universal framework for analyzing technology acceptance theories, which enhances the applicability of the extended AIDUA model across diverse contexts. Furthermore, by considering individual background characteristics, our study systematically explores the deeper mechanisms influencing technology acceptance behaviors within the AIDUA extension model. This exploration reveals how individual differences shape user experiences with artificial intelligence technologies. In summary, this research not only offers a novel theoretical framework for elucidating the acceptance mechanisms of complex AI systems but also establishes a robust foundation and a clear trajectory for the further development and expansion of the AIDUA model.

#### 7.1.2 Constructing the cognition-affect-behavior three-stage model

This study proposes and empirically validates the Cognition-Affect-Behavior three-stage model, exploring the critical role of emotional responses in the acceptance process of GenAI. Unlike traditional TAMs, which emphasize a direct mechanism from cognition to behavior, our findings reveal that emotions—particularly negative emotions—serve as a significant mediating factor between cognitive elements such as performance expectancy and effort expectancy, and users' intention to utilize the technology.

This discovery not only addresses the existing literature's oversight of emotional factors but also provides a novel analytical framework for understanding user decision-making in the context of highly complex technologies. By integrating emotional considerations into the traditional cognitive-behavioral model, this research enhances our comprehensive understanding of the technology acceptance process and underscores the importance of emotional reactions in users' interactions with GenAI technologies.

#### 7.1.3 Shifting evaluation paradigms in pure AI interaction environments

This study reveals the unique acceptance model of GenAI within purely artificial intelligence interaction environments. Unlike traditional artificial intelligence services that emphasize “human-machine collaboration,” users in pure AI systems adopt an absolute evaluation strategy, directly assessing the capabilities and value of the system. The research findings indicate that even when either functionality or hedonic enjoyment is high, users still maintain an independent evaluation of the other aspect of system performance, demonstrating a clear “function-hedonic decoupling” effect.

The “function-hedonic decoupling” effect arises from an in-depth structural analysis of the intrinsic attributes of artificial intelligence technology. Unlike traditional technologies characterized by singular functional attributes, AI tools exhibit a composite set of multiple functionalities and emotional values. This paradigm shift in technology transforms the user acceptance pathway for artificial intelligence products from linear evaluation to multidimensional dynamic trade-offs. From the perspective of theoretical extension, this finding constitutes a substantial supplement to the mainstream theoretical frameworks in technology acceptance research. Traditional TAMs (TAM, UTAUT etc.) assume a singular, linear relationship between functional utility and technology acceptance. The “function-hedonic decoupling” effect proposed in this study does not merely negate existing theories; rather, it offers a more nuanced interpretation of the influencing mechanisms at an ontological level. By clarifying the relative independence of functional value and hedonic experience, this research provides a more complex and dynamic explanatory paradigm for technology acceptance theories.

### 7.2 Practical implications

#### 7.2.1 Enhancing technical transparency to build trust and acceptance

The findings highlight that technical transparency is the most critical factor influencing users' performance expectancy. System designers should prioritize enhancing system explainability and operational transparency by incorporating features that display decision rationales, information sources, and reasoning processes. Such design strategies are particularly crucial in high-stakes domains such as healthcare, law, and finance, where greater transparency can significantly boost user trust and perceived usefulness.

#### 7.2.2 Reducing negative emotions through emotional design

The study reveals that negative emotions act as a critical bridge between user expectations and behavioral intentions. Designers should adopt a “dual-track strategy” that addresses both cognitive and emotional aspects. This includes optimizing system responsiveness, accuracy, and interaction fluidity to alleviate user anxiety. Additionally, providing features such as gradual learning mechanisms, intelligent prompts, and error recovery support can effectively reduce cognitive load, thereby increasing users' willingness to adopt the system.

#### 7.2.3 Implementing differentiated design based on user characteristics

The findings indicate significant differences in generative AI acceptance across user groups. For example, female users exhibit higher sensitivity to anthropomorphic design, older users rely more on system compatibility, highly educated users prioritize technical transparency, and users from different professional backgrounds have distinct evaluation frameworks. System developers should consider adopting adaptive interfaces, customizable interaction modes, and context-aware functionalities to dynamically adjust transparency, anthropomorphism, and assistive features based on users' individual characteristics. This approach can improve acceptance and user experience across diverse user groups.

## 8 Conclusion

### 8.1 Summary

This study, grounded in the perspectives of technological and social attributes, integrates the AIDUA model with cognitive appraisal theory to construct and validate a comprehensive model of GenAI acceptance. By introducing three key variables—technical transparency, system compatibility, and perceived human-AI interaction—this study extends the explanatory power of the AIDUA model. Furthermore, leveraging cognitive appraisal theory, the study proposes a Cognition-Affect-Behavior chain model, systematically elucidating the critical mediating role of emotions between users' cognitive evaluations and behavioral intentions. This multi-perspective integration not only enriches technology acceptance theory but also paves new pathways for understanding user acceptance mechanisms in the context of complex technologies.

Based on an empirical survey of 968 AIGC users, the study yields the following key conclusions: (1) System characteristics (technical transparency, system compatibility, and perceived human-AI interaction) exert significantly stronger effects than social influence, indicating that users' evaluations of generative AI are primarily based on intrinsic technological attributes rather than external social references. Performance expectancy and effort expectancy significantly affect usage intention through the mediating role of negative emotions, highlighting the importance of emotional factors in the acceptance of complex technologies. Users adopt a dual cognitive appraisal mechanism, weighing system benefits against cognitive costs when making acceptance decisions. This finding extends the application of cognitive appraisal theory to the field of technology acceptance. (2) Demographic characteristics exhibit systematic moderating effects in the GenAI acceptance process, revealing significant differences in how various user groups perceive and accept generative AI. These findings provide important insights for personalized design and targeted promotion strategies.

### 8.2 Research limitations and future directions

While this study provides valuable insights into the acceptance mechanisms of GenAI, it has several limitations that warrant further exploration.

#### 8.2.1 Cross-sectional design and dynamic user attitudes

This study employs a cross-sectional design, which limits its ability to capture the dynamic evolution of user acceptance attitudes. Given the rapid iterative development of GenAI technologies, future research should adopt longitudinal designs to track how user evaluation frameworks evolve alongside system capabilities. Particular attention should be paid to the temporal changes in the influence of key factors such as technical transparency and anthropomorphism.

#### 8.2.2 Cultural specificity of the sample

This study focuses on a sample of Chinese users, which may introduce cultural specificity that limits the generalizability of the findings. Considering the cultural differences in AI technology acceptance, future research should expand to cross-cultural comparative studies. For instance, exploring how cultural values (e.g., uncertainty avoidance, collectivism) moderate the impact of system characteristics on user evaluations could help develop a GenAI acceptance model with cross-cultural applicability.

#### 8.2.3 Scenario-specific acceptance mechanisms

The current study does not delve deeply into the differences in acceptance mechanisms across various application scenarios. Generative AI may activate distinct user evaluation frameworks in contexts such as creative content generation, information retrieval, and decision support. Future research should segment application scenarios and investigate how task characteristics (e.g., risk level, complexity, and time sensitivity) moderate the relationships between system characteristics and user expectations, providing theoretical guidance for scenario-specific system design.

#### 8.2.4 Limited focus on multimodal generative AI systems

This study primarily examines GenAI acceptance using ChatGPT as a representative system, without fully considering the unique features of multimodal generative AI systems (e.g., text-to-image or text-to-video generation). Future research should explore the acceptance mechanisms of multimodal GenAI, examining how multimodal output characteristics influence users' cognitive evaluation processes and emotional experiences. This would further enrich the theoretical understanding of GenAI acceptance.

#### 8.2.5 Sampling limitations and digital divide exploration

This study acknowledges a significant sampling limitation characterized by a pronounced educational demographic skew, with 88.1% of participants holding a bachelor's degree or higher. While this sample composition may constrain the external validity and generalizability of the findings, it simultaneously reflects the contemporary phenomenon of digital stratification within the Chinese context. From a methodological perspective, this sampling bias introduces potential construct-level constraints that necessitate careful interpretation of the results. Future research should adopt more sophisticated sampling strategies, including the implementation of stratified random sampling techniques, the development of adaptive survey instruments, and the conduct of comparative analyses across diverse educational demographics. Theoretically, this limitation offers an opportunity to explore the nuanced interactions between technological adoption, educational background, and digital literacy. Subsequent studies could leverage these insights to develop more comprehensive theoretical frameworks that account for variations in technology acceptance behaviors influenced by socio-educational factors.

## Data Availability

The original contributions presented in the study are included in the article/supplementary material, further inquiries can be directed to the corresponding author.
